# Mathematical Model of ATM Activation and Chromatin Relaxation by Ionizing Radiation

**DOI:** 10.3390/ijms21041214

**Published:** 2020-02-12

**Authors:** Yongfeng Li, Francis A. Cucinotta

**Affiliations:** 1 Division of Space and Life Sciences, USRA (Retired), Houston, TX 77574, USA; yfli2004@gmail.com; 2 Department of health Physics and Diagnostic Sciences, University of Nevada Las Vegas, Las Vegas, NV 89154, USA

**Keywords:** Ionizing radiation, oxidative stress, double strand break repair, ATM signaling pathway, chromatin relaxation, biophysics models, systems biology, space radiation

## Abstract

We propose a comprehensive mathematical model to study the dynamics of ionizing radiation induced Ataxia-telangiectasia mutated (ATM) activation that consists of ATM activation through dual mechanisms: the *initiative* activation pathway triggered by the DNA damage-induced local chromatin relaxation and the *primary* activation pathway consisting of a self-activation loop by interplay with chromatin relaxation. The model is expressed as a series of biochemical reactions, governed by a system of differential equations and analyzed by dynamical systems techniques. Radiation induced double strand breaks (DSBs) cause rapid local chromatin relaxation, which is independent of ATM but initiates ATM activation at damage sites. Key to the model description is how chromatin relaxation follows when active ATM phosphorylates KAP-1, which subsequently spreads throughout the chromatin and induces global chromatin relaxation. Additionally, the model describes how oxidative stress activation of ATM triggers a self-activation loop in which PP2A and ATF2 are released so that ATM can undergo autophosphorylation and acetylation for full activation in relaxed chromatin. In contrast, oxidative stress alone can partially activate ATM because phosphorylated ATM remains as a dimer. The model leads to predictions on ATM mediated responses to DSBs, oxidative stress, or both that can be tested by experiments.

## 1. Introduction

Ataxia-telangiectasia mutated (ATM) is a serine/threonine protein kinase that plays a key role in the chromatin remodeling and activation of cell cycle checkpoint in response to the DNA double strand breaks (DSBs) induced by ionizing radiation (IR). Upon activation ATM functions as an upstream signaling protein that phosphorylates a variety of substrates, such as H2AX, p53, Chk2, ATF2, and KAP-1, that are important for the DNA damage response and cell cycle control. Experimental studies have shown that ATM activation involves the *in trans* autophosphorylation and the acetylation by Tip60 and is induced by the MRN complex consisting of the proteins MRE11, RAD50 and NBM, oxidative stress, DSB, and so on. The initiation of ATM activation, however is not fully understood.

Ataxia telangiectasia mutated (ATM) is a protein kinase of the PIKK superfamily, which mediates cellular response to DNA damage by regulating large number of substrates including ATF2, CHK2, KAP-1, MDM2, NBS1, p53, and so on. Many important signaling pathways, such as the NFκB [1] and p53/MDM2 [2] pathways, have been identified to be regulated by ATM. In addition, there is a controversy on the role of ATM in the repair of complex DNA damage [3,4] and DNA damage in the heterochromatin whose compact structure is a barrier blocking the access of DNA repair protein to the damage site [4]. As an upstream signaling protein, ATM plays a crucial role in the DNA damage response, repair pathways, and cell cycle arrest; and how ATM is activated is important for the downstream signaling pathway in response to DNA damage.

Experiments have revealed some but not all of the mechanisms of ATM activation, which are still under debate. It is known that ATM stays in the form of inactive dimer prior to cellular radiation and becomes active as monomer, suggesting that ATM activation is closely related to the dimer-to-monomer transition. It has been proposed that ATM may be activated by oxidative stress [5,6], DNA double strand breaks (DSB) with the help of MRN complex [7], and damage induced chromatin structure change [8]. Meanwhile ATM may undergo autophosphorylation [8] and acetylation by Tip60 [9] during its activation. It is not clear whether the phosphorylation is the cause of or the result of the ATM activation. Recent studies [8] revealed that ATM autophosphorylation in Ser1981 (Ser1987 in mouse) is dispensable for ATM activation suggesting that the autophosphorylation of ATM in Ser1981 is not crucial in the ATM activation. While the block of Tip60 activation inhibits ATM activation, indicating that the acetylation, but not the phosphorylation, of ATM is required for its activation.

Mathematical models have played both descriptive and predictive roles in understanding biological signaling pathways. Approaches to modeling include both deterministic or stochastic approaches [10,11]. We have developed models of the non-homologous end-joining pathway (NHEJ), including a stochastic model of the Ku70/80 heterodimer binding to DNA fragments of varying lengths [12], studies of the order of repair protein binding in NHEJ [13], activation of γH2AX in DSB repair [14], and the role of simple and complex DSBs in repair [14,15]. In spite of the intensive experimental study, little work has been done about the mathematical modeling of ATM activation, which plays a role in both NHEJ and homologous recombination repair. There are several models which use over-simplified ATM activation mechanisms, however whose purposes are not to study ATM activation but for example the detection mechanism of DSB [16] or the dynamics of p53 and NFκB in response to DNA damage [17,18,19].

In this work, we proposed a comprehensive kinetic model of ATM activation whereby ATM is activated by two distinct pathways. One is the *initiative* activation pathway triggered by the DNA damage-induced local chromatin relaxation that is independent of ATM [20]. The other is the *primary* activation pathway consisting of a self-activation loop by interplay with chromatin relaxation. Active ATM phosphorylates KAP-1 at the DNA damage site from which phosphorylated KAP-1 enhances local chromatin relaxation and spreads throughout the nucleus to convey the signal for global chromatin relaxation [21]. Under the help of MRN, ATM is recruited to the damage site [7]. Active ATM phosphorylates ATF2 so that Tip60 is activated by binding with H3K9me3 in the relaxed chromatin to acetylate ATM [22,23,24]. Meanwhile, ionizing radiation or other damaging agents induce reactive oxygen species (ROS), which removes the inhibition of PP2A [25] so that ATM undergoes autophosphorylation [26]. Phosphorylation and acetylation of ATM together lead to the ATM dimer-to-monomer transition for full ATM activation. As discussed above, several simple models of ATM activation have appeared in the scientific literature [16,17,18,19]. However, in the present work we show, for the first time, that a detailed mathematical model is constructed accordingly to describe the biochemical reactions involving in the ATM activation. The important predictive tool that results from the model is demonstrated by the kinetic profiles that are revealed by numerical simulation of the model under radiation.

## 2. Results

### 2.1. Model Development

ATM stays inactive as a dimer in normal and many transformed cells. When exposed to radiation, ATM becomes an active monomer to phosphorylate the subsequent substrates. It is still under debate about the mechanism of ATM activation. It has been proposed that the ATM activation may involve ATM autophosphorylation [8], acetylation by Tip60 [24], MRN functioning [7], or chromatin structure change [8]. However, the ATM monomer can be kinase active even though it is not phosphorylated [8]. Therefore, the description of ATM activation and the mechanisms of its dimer-to-monomer transition is the goal of our model development.

**Figure 1:**
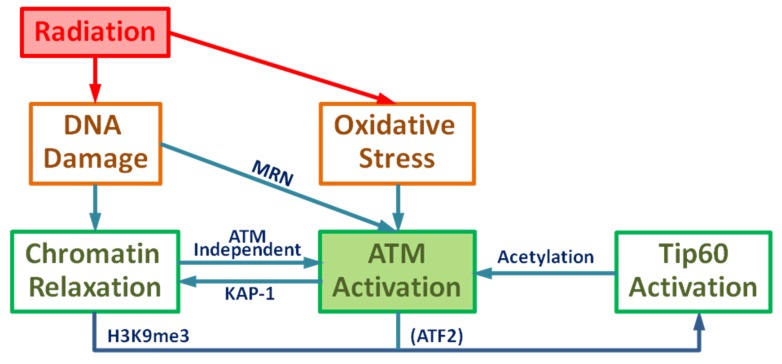
Schematic diagram of Ataxia-telangiectasia mutated (ATM) activation: Ionizing radiation leads to DNA damage and oxidative stress resulting in the activation of ATM and chromatin relaxation.

Radiation causes DNA damage including DSBs through direct ionization and the indirect effect of ROS induction or combination of these two damage mechanisms, which can affect various signal transduction pathways. DSBs cause local chromatin relaxation, triggering ATM activation by the initial activation pathway. Active ATM phosphorylates KAP-1 at the DNA damage site and phosphorylated KAP-1 spreads out throughout nucleus and induces global chromatin relaxation. Tip60 is activated by ATM-dependent phosphorylation of ATF2 and binding to H3K9me3 in the relaxed chromatin where the histone binding protein HP1 is removed by CK2 phosphorylation to release H3K9me3. Consequently, the DNA damage by IR results in the ATM acetylation by Tip60. Meanwhile, the general induction of ROS removes PP2A resulting in ATM autophosphorylation. Therefore, these two biological effects of IR contribute to ATM acetylation and phosphorylation and results in the ATM transition from dimer to monomer, leading to full ATM activation by the primary activation pathway. Moreover, chromatin relaxation is enhanced in the ATM-dependent manner. As an outline, a complete diagram of full ATM activation is given in Figure 1.

**Figure 2:**
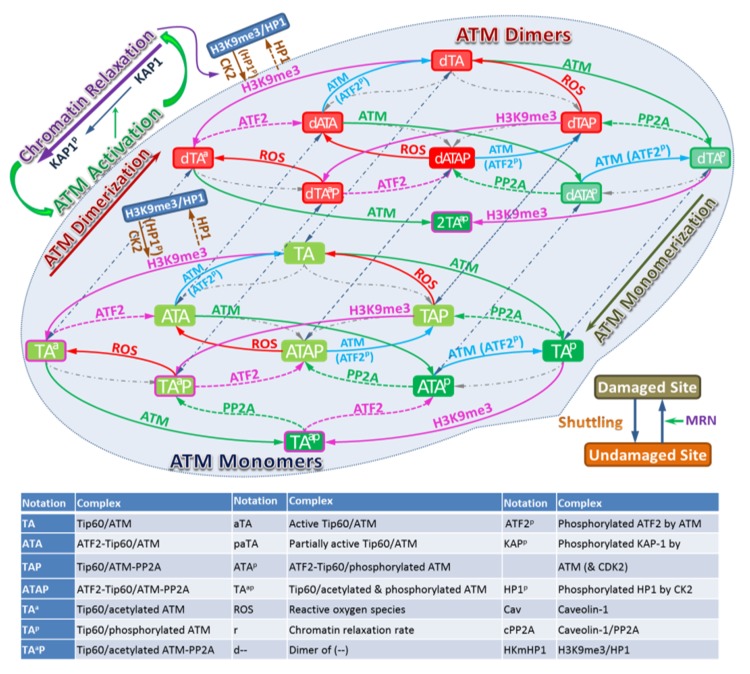
Schematic description of the model for ATM Activation. The left panel considers undamaged chromatin and the middle panel damaged chromatin. Biochemical species included in the model are indicating by the Legend in Figure 2 with arrows illustrating interactions included in the model.

The model we develop uses the method of coupled ordinary differential equations to represent biochemical reactions as described in the Methods section and Supplementary Material. A detailed working model of ATM activation is illustrated in Figure 2, which includes the following steps:

(1) Radiation causes DNA damage and the production of ROS;
(2) DNA damage induces local chromatin relaxation, which
(3) Triggers ATM activation (dimer-to-monomer transition);
(4) ROS removes PP2A from ATM for ATM autophosphorylation (partially activated ATM–autophosphorylated ATM dimer);
(5) Active ATM phosphorylates ATF2 for its release from Tip60;
(6) Active ATM phosphorylates KAP-1 at DNA damage site and
(7) Phosphorylated KAP-1 enhances local chromatin relaxation and conveys signal for global chromatin relaxation;
(8) HP1β is phosphorylated by CK2 in the relaxed chromatin to be released from H3K9me3;
(9) Tip60 binds with H3K9me3 to activate its HAT activity;
(10) ATM is acetylated by Tip60 and autophosphorylated for its dimer-to-monomer transition;
(11) ATM is shuttling between damaged and undamaged sites and MRN enhances its recruitment to the damaged site.

**Figure 3:**
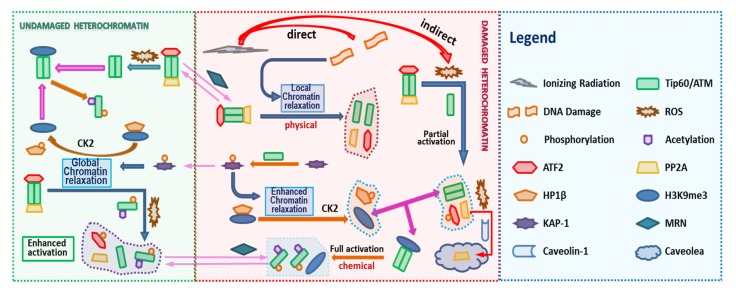
Biochemical Reactions in the ATM Activation. ATM activation consists in a series of biochemical reactions, protein binding/unbinding, including phosphorylation/dephosphorylation, acetylation/deacetylation. The Figure 2 Legend lists names of molecules considered in the reaction pathway. In addition, the shuttling of molecules between the DNA damaged site and undamaged site can be written in terms of appropriate reactions (See Supplementary Material).

ATM stably associates with Tip60, therefore in the model we assume that ATM and Tip60 form a stable complex that is treated as a single entity. The associated biochemical reactions are illustrated in Figure 3. In Figure 2 and Figure 3 the arrows have the usual meaning used in chemical kinetics descriptions where the arrow points in the direction of the reaction, while two-way arrows refer to reversible reactions. The center panel of Figure 3 shows both direct and indirect effects are sources for ATM activation, however differences in these two pathways are described by the model. Additional discussion about the modeling is provided below and more details are given in the Supplementary Material. The mathematical model of these reactions leads to a large stoichiometric matrix. In the Supplementary Material we show how to reduce the large stoichiometric matrix to a modular form.

#### 2.1.1. ATM Activation—Dimer to Monomer Transition

In the model, we assume that there are dual mechanisms of inducing the ATM dimer-to-monomer transition. One is due to changes in chromatin structure. DNA is packed and coiled into a high order structure compactly to form heterochromatin, which requires energy. In other words, potential energy has been deposited into highly compacted heterochromatin to maintain its condensed structure. Once exposed to radiation to cause DNA breaks, the compact structure in the vicinity of breaks collapses to release the energy leading to rapid chromatin structure change locally. This local chromatin relaxation induced by radiation has been observed in yeast [27] and mammalian cells [12] and may be a very rapid process independent of ATM [12]. Such rapid local chromatin relaxation may cause the breakage of the ATM dimer binding, resulting in ATM dimer dissociation to monomer. Thus ATM activation by chromatin structure change is more likely a physical activation and is independent of ATM initially. Nevertheless, chromatin relaxation also depends on ATM and KAP1, which leads to global chromatin relaxation and will be discussed shortly. Therefore, we suggest that radiation induces rapid ATM-independent local chromatin relaxation followed by ATM-dependent relaxation which is slow because ATM activation is required. This is consistent with the experimental observation that ‘’the behavior of chromatin following DNA breakage exhibits a period of fast relaxation followed by a period of slower relaxation” [28]. In addition, such activation should be distinguished from ATM activation by ATP [9] that is dependent on ATM autophosphorylation. Here we call it the *initiative ATM activation pathway*.

The other ATM activation pathway is through the ATM autophosphorylation and acetylation by Tip60. Phosphorylated ATM still remains as a dimer [5] and solely acetylated ATM is not kinase active, therefore it is reasonable to assume that both autophosphorylation and acetylation are required for ATM dimer-to-monomer transition for full ATM activation. In comparison with chromatin relaxation induced ATM activation, ATM activation through autophosphorylation and acetylation is a chemical activation to induce ATM conformal change leading to the dimer dissociation. As a result of the dependence of chromatin structure, this secondary activation pathway triggers a positive feedback loop to enhance ATM activation in a dominate way. Thus we call it the *primary ATM activation pathway*.

#### 2.1.2. ATM Acetylation—Tip60 Activation and H3K9me3

It is reported that ATM activation requires ATM acetylation by Tip60 [24]. Independent of DNA damage, ATM forms a stable complex with Tip60 through the conserved FATC domain of ATM and hence they are recruited to the DSBs simultaneously [9]. Tip60 is inhibited by binding with ATF2 via the MYST domain and their interaction is diminished after ionizing radiation [22]. Free Tip60 binds to H3K9me3 to activate its HAT activity. Subsequently ATM is acetylated by activated Tip60. ATF2 is a substrate of ATM and its phosphorylation by ATM is required for DNA damage response [23]. Thus it is reasonable to assume that ATF2 is phosphorylated by ATM to cause its conformal change leading to its dissociation from Tip60. On the other hand, heterochromatin is highly compacted to prevent molecules from binding to histones. Here chromatin relaxation is needed so that H3K9me3 is exposed for binding with Tip60. Therefore, Tip60 activation replies on the chromatin relaxation.

H3K9me3 is predominantly located in the compacted heterochromatic regions where it is associated with members of the chromatin protein (HP1) family [29]. Hence the ability of Tip60 to access H3K9me3 is dependent on the DNA damage induced displacement of HP1β from H3K9me3 [24]. Although it is assumed that CK2 is activated by unknown mechanism to phosphorylate HP1β so that HP1β is released from H3K9me3, it is proposed that CK2 is always active within the cell [30]. In the model, therefore, we assume that CK2 is always active but cannot reach histone to phosphorylate HP1 unless the chromatin is relaxed.

#### 2.1.3. ATM Autophosphorylation—PP2A and ROS

It was observed that ATM autophosphorylation is regulated by PP2A [26]. In the absence of DNA damage, dimers of ATM undergo autophosphorylation (at serine 1981) that is removed by bound PP2A, showing the inhibition of PP2A on ATM autophosphorylation. On the other hand, under the oxidative stress, PP2A translocates into caveolae membranes and interacts with Caveolin-1 [25], which interferes with the binding of PP2A to ATM. Consequently, this provides a clue that oxidative stress inhibits the activity of PP2A on ATM so that ATM undergoes autophosphorylation, which is consistent with experimental observations [5,6].

In addition to serine 1981, other phosphorylation sites have been identified, such as serine 367, serine 1893 [31] and serine 2993 [32]. However, it not clear if they are distinct functions for the various phosphorylation sites other than that several function in auto-phosphorylation. In the model, we take consider serine 1981 into account, which can be viewed as a surrogate for sites that lead to activation. Further discussion of this point is made below.

#### 2.1.4. ATM-Dependent Chromatin Relaxation

It has been shown that DSB formation is followed by ATM-dependent chromatin relaxation [21]. KAP-1, the KRAB-associated protein, is a novel ATM substrate in the DNA-damage response. As suggested in [21], KAP-1 is phosphorylated exclusively at the DNA damage sites, from which phosphorylated KAP-1 spreads rapidly throughout the chromatin, leading to global chromatin relaxation. However, it is unknown how KAP-1 conveys the signal of chromatin relaxation. On the other hand, it has been shown that two E3 ubiquitin ligases, RNF8, and Chfr, synergistically regulate histone ubiquitination to control histone H4K16 acetylation for chromatin relaxation, and that the deficiency of RNF8 and Chfr inhibits DN damaged induced ATM activation, indicating a model that RNF8 and Chfr are important for histone ubiquitination and acetylation that in turn induce chromatin relaxation, which facilitates ATM activation [33]. Therefore, it is suggested that chromatin relaxation and ATM activation enhance mutually and form a positive feedback loop.

For the sake of studying the ATM activation, in this model, we ignored the details of chromatin relaxation related to RNF8, Chfr, KAP-1, etc. Instead, we introduce a quantity r, which is called the relaxation rate of the chromatin and ranges from 0 to 1. Namely the chromatin is fully compact if r = 0 and completely relaxed as r = 1. Thus the chromatin structure change is characterized by the change in the rate of the relaxation rate r. We assume that the relaxation rate depends only on the KAP-1 and phosphorylated KAP-1 (pKAP-1). Precisely KAP-1 promotes chromatin condensation, while pKAP-1 promotes the chromatin decondensation (relaxation). Therefore, pKAP-1 enhances the local chromatin relaxation induced by DSBs at the chromatin damaged site, and pKAP-1 spread throughout the nucleus accounts for the global chromatin relaxation at the undamaged site. This ATM-dependent chromatin relaxation is a subsequent response, following the ATM-independent relaxation, to the DNA damage.

#### 2.1.5. Partial ATM Activation by ROS

Without the presence of DSBs, ROS cannot activate ATM fully. As reported in [5], ATM autophosphorylation at Ser1981 occurs following H_2_O_2_ treatment. This is because ROS removes PP2A from ATM and blocks its inhibition to ATM autophosphorylation, as discussed above. However, phosphorylated ATM remains in the form of dimer and can phosphorylate only part of substrates, such as p53 and Chk2 but not γ-H2AX and Kap1, of fully active ATM (Figure 3). Therefore, consider the phosphorylated ATM dimer as partially activated ATM.

#### 2.1.6. The Role of MRN in the ATM Activation

It is believed that MRN complex plays key role in the ATM activation [7,34,35]. It is observed, however, that ATM dimer-to-monomer transition occurs under the high concentration of DSBs without the presence of MRN [36,37,38], indicating that MRN is not essential to the ATM activation. Therefore, we assume that MRN plays the role of DSB sensor for ATM that helps ATM bind to DSBs especially in the low concentration of DSBs, while this role of DSB sensor is significantly weakened as DSBs are in high concentration.

#### 2.1.7. Full ATM Activation by IR and Bleomycin

Bleomycin (BLM) is a radiomimetic anticancer drug that can generate DSBs [7] and causes an increase of ROS level [8,39]. Therefore, Bleomycin may activate ATM in the similar manner as ionizing radiation. It is reported in [5] that ROS-induced ATM activation is followed by the phosphorylation of only p53 and CHK2, while Bleomycin-induced ATM activation also leads to the phosphorylation of γ-H2AX and Kap1 additionally, which is shown in Table S1. In other words, Bleomycin induces full ATM activation as IR does.

### 2.2. Numerical Simulation

In the following, we provide some numerical simulation of the model. The model contains over 20 molecular species as detailed in the Supplementary Material. There is little knowledge of all the parameters employed in the model and sparse experimental data for comparison, therefore we assume that all the parameters are dimensionless and only show the kinetic profile of the model using roughly estimated values of the parameters. We assume the acute dose of radiation whose dose rate is $D_{R}=1$ and exposure time is T=1.

**Figure 4:**
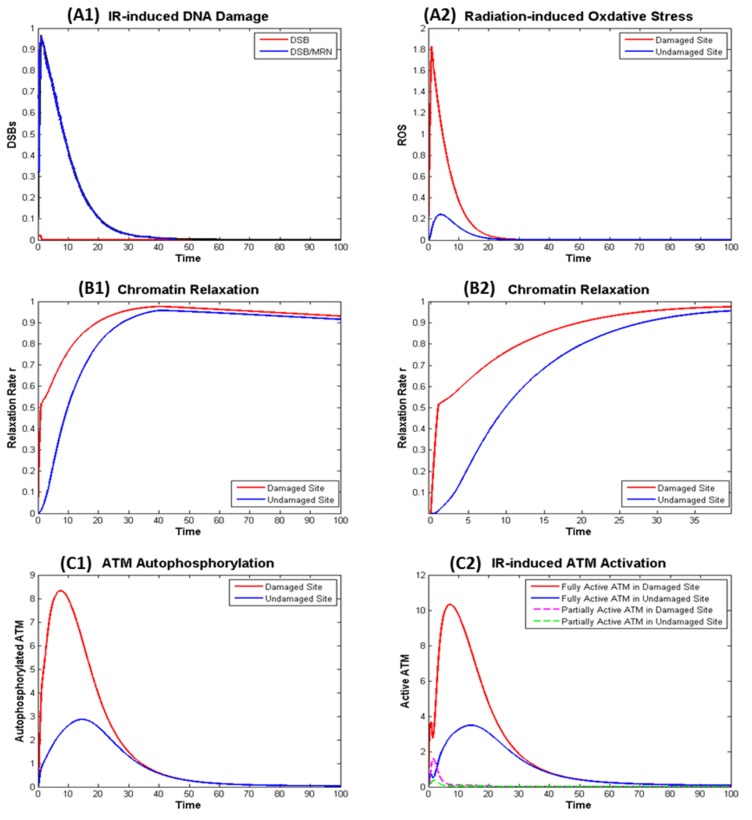
Numerical simulation of the dynamics in the ionizing radiation (IR) induced ATM Activation in the Damaged and Undamaged Sites. (**A**) Effect of radiation: (**A1**) direct effect—DNA Damage; (**A2**) indirect effect—Oxidative Stress; (**B**) IR induced chromatin: (**B1**) kinetics of chromatin relaxation within t = 100; (**B2**) kinetics within t = 40 in which rapid relaxation within around t = 1 can be observed. (**C**) IR induced ATM autophosphorylation (**C1**) and (partial and full) ATM activation (**C2**).

Exposure to the radiation leads to rapid generation of DSB (about 30 DSB per Gy) and ROS that are later repaired and removed shown in a normalized manner in Figure 4 panels A1 and A2. The generation of DSB induces fast chromatin relaxation followed by slow relaxation after radiation ceases as exhibited in Figure 4 panels B1 and B2, consistent with the observations from experiment [28]. After DSBs are completely repaired, chromatin condensation starts slowly.

Figure 4 shows IR induced ATM autophosphorylation (left panel C1), that is not exactly the same as the active ATM (right panel C2). This is because phosphorylated ATM may not be fully activated if it remains as dimer and ATM monomer is fully activated even if it is not phosphorylated. The model as described in the Supplementary File describes the kinetics of KAP-1, pKAP-1, MRN, Tip60, etc. and can be used to model the kinetics of the γH2AX, which is often used as beacon for DSB induction and removal, in a similar manner as our earlier work [14].

## 3. Discussion

The serine/threonine protein kinase ataxia telangiectasia mutated (ATM) plays a critical role in the DNA damage response, including roles in DSB repair pathway choice and its absence leads to extreme radiation sensitivity [40,41]. In this report we present a detailed mathematical model of ATM activation following irradiation and ROS. The model focuses on observations of DSB repair heterochromatin [4,42,43,44,45]. A role for ATM in relaxation of chromatin in euchromatin is observed to be less important in experiments [4,40]. Figure 2 and Figure 3 display a very detailed model with over 20 molecular species, while Figure 1 shows a reduced schematic model which can serve as a basis for a parametric model calibrated with experimental data, which was not the focus of the present work.

It has been reported that ATM activation is related to MRN [7,34,35], ATM autophosphorylation [8] and ATM acetylation by Tip60 [22,24], ROS [6], and chromatin structure change [8]. However, it is not well understood how these factors cooperate in activation of ATM. In this work, we assume that the full ATM activation means ATM dimer-to-monomer transition is through a dual activation mechanisms. One is the *initiative activation* pathway triggered by the ATM-independent local chromatin relaxation, the other is the *primary activation* pathway to enhance the activation, resulting from Tip60 activation to acetylate ATM and ATM autophosphorylation by removing the PP2A inhibition by ROS.

Precisely, exposed to ROS, PP2A is released from ATM and bound to the membrane protein Caveolin. The inhibition of PP2A promotes ATM autophosphorylation. However, ATM remains as a dimer under the ROS-induced autophosphorylation. Such dimeric phosphorylated ATM can phosphorylate only a portion of the substrates, say p53 and CHK2, that fully activated ATM phosphorylation targets. Thus such dimeric phosphorylated ATM is called partially activated ATM. This distinguishes ATM activation induced by ROS and IR. Moreover, Bleomycin, another ATM activation inducer, can produce both DSB and ROS, thus it can fully activate ATM like IR does. Therefore, the model applies to the ATM activation induced by all the agents that we have introduced. Our model considers ATM autophosphorylation at serine 1981. However, a large number of sites have been identified [32,33,46] albeit there precise role in ATM activation has not been elucidated. The model equations could be expanded to include other sites resulting in a larger system and stoichiometric matrix, however in the current model we may consider the role of serine 1981 as a surrogate for a group of sites participating in auto-phosphorylation.

In addition, the initiative activation pathway relies on chromatin relaxation, which is crucial for the DSB repair in the heterochromatin. In the euchromatin, however, chromatin structure change is not significant and consequently it is not clear whether the relaxation of euchromatin is able to trigger ATM activation. On the other hand, because of the loose structure, euchromatin is relaxed in an ATM-independent way, that differs from the heterochromatin relaxation. Although H3K9me3 may be accessible to the Tip60 for its activation in the loose euchromatin, active ATM is still required to phosphorylate and release ATF2 from Tip60. From this viewpoint, the proposed model seemingly applies only to the ATM activation in the heterochromatin region. Therefore, it is suggested by the model that ATM activation starts in the heterochromatin and spreads out into euchromatin. We expect that the dual mechanisms tested here and the resulting mathematical models can be investigated experimentally through the study of agents that induced differential amounts of DNA damage and ROS, such as comparisons of high to low linear energy transfer radiation [47,48].

## 4. Materials and Methods

### 4.1. Biochemical Reactions

According to the working model provided in Figure 1, the ATM activation can be described as a system of biochemical reactions of ATM and other participating proteins, which has been summarized and illustrated in Figure 2 and Figure 3. The initiative activation pathway through chromatin relaxation is governed by

$$\mathrm{ATM}\;\mathrm{dimer}\;\overset{\mathrm{chromatin}\;\mathrm{relaxation}}{\to }\;2\;\mathrm{ATM}\;\mathrm{momoners}$$

In the damaged chromatin site, chromatin relaxation depends on not only the phosphorylated KAP-1, but also the DNA damage which dominates the immediate damage response. With the presence of ROS and relaxed chromatin, the primary ATM activation pathway involves the following major reactions

$$\mathrm{Tip}60/\mathrm{ATF}2\overset{\mathrm{active}\;\mathrm{ATM}}{\to }\mathrm{Tip}60+\mathrm{pATF}2,\;\mathrm{Tip}60+H3K9\mathrm{me}3\overset{r}{\to }\mathrm{Tip}60/H3K9\mathrm{me}3,\;\mathrm{dimer}\;\mathrm{ATM}/\mathrm{PP}2A\overset{\mathrm{ROS}}{\to }\mathrm{dimer}\;\mathrm{pATM}+2\;\mathrm{PP}2A,\;\mathrm{dimer}\;\mathrm{pATM}\overset{\mathrm{Tip}60/H3K9\mathrm{me}3}{\to }2\;\mathrm{apATM}\;\mathrm{monomers}.$$

All other reactions can be written in the similar way, and more details can be found in the Supplementary Material.

### 4.2. Mathematical Modeling of ATM Activation

With prescribed kinetics of DNA damage, chromatin relaxation and ROS production as the external control u and by the law of mass action or Michaelis-Menten kinetics, ATM activation can be modeled in terms of a system of ordinary differential equations of the following form

$$\frac{dX_{0}}{dt}=SR_{0}\left(X_{0},u_{0}\right)-SH\left(X_{0},X_{1}\right)$$

$$\frac{dX_{0}}{dt}=SR_{1}\left(X_{1},u_{1}\right)+SH\left(X_{0},X_{1}\right)$$

where S is the stoichiometric matrix, $R_{i}$ the reaction flux vector, $X_{i}$ the reacting species (state variable including ATM, associated proteins and their complex in the forms of monomer and dimer), with the damaged site for i=0 and undamaged site for i=1 ; and $\mathrm{SH}\left(X_{0},X_{1}\right)$ indicates the shuttling of all the reacting species between the two sites. Obviously the controls applied to the two sites are distinguished by the presence of DNA damage, that is, $u_{0}=\left(\mathrm{DSB},{\;r}_{0},{\mathrm{ROS}}_{0}\right)$ and $u_{1}=\left(r_{1},{\mathrm{ROS}}_{1}\right)$. Extensive details about the model can be found in the Supplementary Material.

### 4.3. Phenomenological Model of the Effects of ATM Activation Triggering Agents

ATM activation can be triggered by different agents such as oxidative stress, Bleomycin, ionizing radiation, etc. By our assumption of modeling, oxidative stress alone does not induce chromatin relaxation and induces only partial ATM activation. Therefore, the prescribed kinetics of ROS level is enough to be the controller u of the ATM activation. In contrast, both Bleomycin (BLM) and ionizing radiation produce ROS and DSBs leading to chromatin relaxation, a kinetic model of BLM or IR induced ROS, DSB, and chromatin relaxation is needed. Such a model may take the follow form

$$\frac{du}{dt}=F\left(u,v;X\right)$$

where v is the triggering agent such as BLM and IR. The dependence of chromatin relaxation on the phosphorylated KAP-1 suggests the dependence of F on X through pKAP-1 as feedback. Instead of non-homologous end joining that is for simple DSB repair and independent of ATM, if we assume the DSB repair is through ATM-dependent NHEJ for complex DSB repair or homologous recombination in which ATM is required, then F depends on X additionally through ATM, showing how the model of triggering agent couples with model of ATM activation. More details have been discussed in the Supplementary Material.

Indeed, accumulated ROS can induce the production of DSB, especially in S-phase or during DNA replication, and sustained unrepaired DSB also induces the production of ROS. In other words, ROS and DSB form a positive feedback loop. Therefore, along with the removal mechanism of ROS and repair mechanism of DSB, the kinetics between ROS and DSB becomes more complex (leading to bistability) than the scenario studied in this paper. The purpose of this paper is to study how ATM is activated, therefore we ignored the positive feedback effect between ROS and DSB.

The dynamical changes of the concentrations of all the participating species in the ATM activation can be simulated by numerically solving the above system of ordinary differential equations, as shown in the Results section. The model takes into account the DNA damaged site and undamaged site separately, therefore the reacting species on the two sites are treated as different species, leading to a larger system. By adding the controlling species, the model system has >100 dimensions. Hence the focus of this work is not on parameter estimation and comparison of the existing sparse experimental. This large system is simulated by an ODE solver (ode45) in the Matlab 7.13 (R2001b).

## Supplementary Materials

Supplementary materials can be found at https://www.mdpi.com/1422-0067/21/4/1214/s1.
