# Supplementary material for: Mathematical Model of ATM Activation and Chromatin Relaxation by Ionizing Radiation
Source: Int J Mol Sci. 2020 Feb 12;21(4):1214. doi: 10.3390/ijms21041214 (PMC7072770; doi:10.3390/ijms21041214)
Supplement: Supplementary file 1 [file ijms-21-01214-s001.pdf]

ATM Activation Induced by Chromatin Relaxation
- Supplemantary Material

1 Reacting Species

In the following, we will introduce some new notations, listed in the Table S1, to represent all the species in the ATM activation. We will explain in details during the modeling how these species come into the play in the ATM activation.

Notation	Species
DSB	DNA double Strand Break
DM	DSB/MRN complex
ROS	reactive oxygen species
r	relaxation rate of chromatin
TA	Tip60/ATM
ATA	ATF2-Tip60/ATM
TAP	Tip60/ATM-PP2A
ATAP	ATF2-Tip60/ATM-PP2A
dATAP	dimer of ATF2-Tip60/ATM-PP2A
aTA	active Tip60/ATM
paTA	partially active Tip60/ATM
KAP1	KAP-1
$KAP1^p$	phosphorylated KAP-1
ATF2	ATF2
$ATF2^p$	phosphorylated ATF2
H3K9me3	H3K9me3
HKmHP1	H3K9me3/HP-1
HP1	HP-1
$HP1^p$	phosphorylated HP-1
PP2A	PP2A
Cav	Cavelion-1
CPP2A	Cavelion/PP2A

Table S1. Notations of Reacting Species in the ATM Activation.

Except DSB and DSB/MRN complex, denoted by DM, we assume that all the species can be either in the DNA damaged site or undamaged site. Consequently

if we treat the same proteins located in the different sites to be different species, then the number of reacting species will be doubled. Moreover, we divide all the reacting species into several groups, denote by

$$
\begin{array}{llcl}
\text{control:} & u & = & (\mathsf{DSB}, \mathsf{DM}, \mathsf{MRN}_0, \mathsf{MRN}_1, \mathsf{ROS}_0, \mathsf{ROS}_1)^\top \\
\text{relaxation rate:} & X_0 & = & (\mathsf{r}_0, \mathsf{r}_1)^\top \\
\text{pure monomers:} & X_1^i & = & (\mathsf{TA}, \mathsf{TA^a}, \mathsf{TA^p}, \mathsf{TA^{ap}})_i^\top \\
\text{bound monomers:} & X_2^i & = & (\mathsf{ATA}, \mathsf{ATA^p}, \mathsf{TAP}, \mathsf{TA^aP}; \mathsf{ATAP})_i^\top \\
\text{pure dimers:} & X_3^i & = & (\mathsf{dTA}, \mathsf{dTA^a}, \mathsf{dTA^p})_i^\top \\
\text{bound dimers:} & X_4^i & = & (\mathsf{dATA}, \mathsf{dATA^p}, \mathsf{dTAP}, \mathsf{dTA^aP}; \mathsf{dATAP})_i^\top \\
\text{other proteins:} & X_5^i & = & (\mathsf{KAP1}, \mathsf{KAP1^p}, \mathsf{HP1}, \mathsf{HP1^p}, \mathsf{ATF2}, \mathsf{ATF2^p}, \mathsf{PP2A})_i^\top \\
\text{other proteins:} & X_6^i & = & (\mathsf{H3K9me3}, \mathsf{HKmHP1}, \mathsf{Cav}, \mathsf{CPP2A})^\top ,
\end{array}
$$

where subscript $i = 0$ indicates all the species on the damaged site, and $i = 1$ those in the undamaged site. Let

$$\mathbb{X}_i = \left(X_1^i, X_2^i, X_3^i, X_4^i, X_5^i\right), \qquad i = 0, 1.$$

Then (u, X_0) and $\mathbb{X} = (\mathbb{X}_0, \mathbb{X}_1)$ give a compact form of all the reacting species in the ATM activation.

2 Modeling the Triggers of ATM Activation

It has been suggested that ATM may be activated by DNA damage and oxidative stress, both of which are the IR effects and directly connect IR with ATM activation in a chromatin-structure dependent manner. In this section, we will model how IR induces DSB and ROS and subsequent chromatin relaxation. They are key components of the initial stage proceeding the ATM activation and play the role of triggers of ATM activation.

2.1 IR Induced DNA Damage and Oxidative Stress

Let $\mathsf{D_R}$ be the radiation dose rate and the total dose is given by $\int_0^\infty \mathsf{D_R}(t)dt$. Suppose that IR induced production rates of DSB and ROS are proportional to dose rate $\mathsf{D_R}$, by rates $\mathsf{b_{DSB}}$ and $\mathsf{b_{ROS}}$, respectively. Once DSBs are generated, MRN quickly recognizes the damage and binds to the DSB at rate $\mathsf{k_a^0}$ and form a complex denoted by DM. On the other hand, we assume that ROS is induced in the damaged site, and either removed at rate $\mathsf{r_{ROS}}$ or diffuses to the undamaged site at rate $\mathsf{d_{ROS}}$. Then the dynamics of the species discussed above can be governed by the following system of differential equations

$$
\boxed{
\begin{aligned}
\frac{d\mathsf{DSB}}{dt} &= \mathsf{b_{DSB}}\mathsf{D_R} - \mathsf{k_a^0}\mathsf{DSB}\,\mathsf{MRN}_0 \\
\frac{d\mathsf{ROS}_0}{dt} &= \mathsf{b_{ROS}}\mathsf{D_R} - \mathsf{d_{ROS}}(\mathsf{ROS}_0 - \mathsf{ROS}_1) - \tfrac{\mathsf{r_{ROS}}\mathsf{ROS}_0}{1+\mathsf{ROS}_0} \\
\frac{d\mathsf{ROS}_1}{dt} &= \mathsf{d_{ROS}}(\mathsf{ROS}_0 - \mathsf{ROS}_1) - \tfrac{\mathsf{r_{ROS}}\mathsf{ROS}_1}{1+\mathsf{ROS}_1}
\end{aligned}
}
\qquad (1)
$$

where Hill's type function $\frac{x}{1+x}$ is used to indicate the saturation in the removal of ROS. Indeed, ROS can also generate DSB and sustained unrepaired DSB can induce the production of ROS, leading to a positive feedback loop between ROS and DSB. In the scenario, the corresponding equations become

$$\left\{\begin{array}{lcl} \frac{d\mathsf{DSB}}{dt} & = & \mathsf{b_{DSB}}\,\mathsf{D_R} - \mathsf{k_a^0}\mathsf{DSB}\,\mathsf{MRN}_0 + \boxed{\mathsf{b_{DR}}\,\mathsf{ROS}} \\ \frac{d\mathsf{ROS}_0}{dt} & = & \mathsf{b_{ROS}}\,\mathsf{D_R} - \mathsf{d_{ROS}}(\mathsf{ROS}_0 - \mathsf{ROS}_1) - \mathsf{r_{ROS}}\,\mathsf{ROS}_0 + \boxed{\mathsf{b_{RD}}\,\mathsf{DSB}} \end{array}\right. \tag{1r}$$

where $\mathsf{b_{DR}}$ is the production rate of DSB by ROS, and $\mathsf{b_{RD}}$ is the production rate of ROS by DSB. In either case, $\mathsf{D_R}$ is the external control input and the resulting DSB and ROS are the output.

2.2 DNA Damage Induced Chromatin Relaxation

It has been revealed in the experiments that once exposed to the radiation, heterochromatin is relaxed very rapidly around the damaged site in the ATM-independent manner, followed by slow relaxation. ATM is activated during the chromatin relaxation, which will be discussed shortly in Section 3.1. In addition, active ATM phosphorylates KAP-1, denoted by $\mathsf{KAP1}$, exclusively in the damage site and phosphorylated $\mathsf{KAP1}$, denoted by $\mathsf{KAP1^p}$, spreads out throughout the nucleus and causes global chromatin relaxation, indicating the slow kinetics in the relaxation process. Thus we assume that chromatin relaxation is enhanced by $\mathsf{KAP1^p}$ but inhibited by $\mathsf{KAP1}$, or chromatin condensation is enhanced by $\mathsf{KAP1}$. Accordingly we have the following model for the chromatin relaxation,

$$\boxed{\begin{array}{lcl} \frac{d\mathsf{r}_0}{dt} & = & \dot{\mathsf{r}}_0 = [f(\mathsf{DSB}+\mathsf{DM}) + g(\mathsf{KAP1^p}_0)]\,(1-\mathsf{r}_0) - h(\mathsf{KAP1}_0)\mathsf{r}_0 \\ \frac{d\mathsf{r}_1}{dt} & = & \dot{\mathsf{r}}_1 = g(\mathsf{KAP1^p}_1)(1-\mathsf{r}_1) - h(\mathsf{KAP1}_1)\mathsf{r}_1 \end{array}} \tag{2}$$

where functions f, g and h are non-negative increasing functions such as Hill's function $\frac{x}{K+x}$, implying that the more DSB or more $\mathsf{KAP1^p}$, the more rapidly the chromatin is relaxed. The missing term f in the second equation shows that DNA damage induced relaxation is exclusively in the damage site.

2.3 R0: Recruitment, Release and Shuttling of MRN

As discussed in Section 2.1, MRN first binds with DSB to form a complex, called DM (R0-1). Moreover, we assume that MRN will be released for reuse after the damage is repaired with rate $\mathsf{r_{DSB}}$ (R0-2). Besides, MRN shuttles between the damaged and undamaged sites (R0-3).

$$\boxed{(1)\ \mathsf{DSB}+\mathsf{MRN}_0 \xrightarrow{\mathsf{k_a^0}} \mathsf{DM},\quad (2)\ \mathsf{DM} \xrightarrow{\mathsf{r_{DSB}}} \mathsf{MRN}_0,\quad (3)\ \mathsf{MRN}_0 \underset{\mathsf{k_{im}^0}}{\overset{\mathsf{k_{ex}^0}}{\rightleftharpoons}} \mathsf{MRN}_1} \tag{R0}$$

Here $\mathsf{k}^0_{\mathsf{im}}$ and $\mathsf{k}^0_{\mathsf{ex}}$ are the import rate (from undamaged site to damaged site) and export rate of MRN, respectively. By the law of mass action, the reaction rate equations of reaction R0 is given in equation (3).

$$\begin{aligned}
\frac{d\mathsf{DM}}{dt} &= \mathsf{k}^0_{\mathsf{a}}\mathsf{DSB}\,\mathsf{MRN}_0 - \mathsf{r}_{\mathsf{DSB}}\mathsf{DM}, \\
\frac{d\mathsf{MRN}_0}{dt} &= \mathsf{r}_{\mathsf{DSB}}\mathsf{aTA}_0\mathsf{DM} - \mathsf{k}^0_{\mathsf{a}}\mathsf{DSB}\,\mathsf{MRN}_0 - (\mathsf{k}^0_{\mathsf{ex}}\mathsf{MRN}_0 - \mathsf{k}^0_{\mathsf{im}}\mathsf{MRN}_1) \\
\frac{d\mathsf{MRN}_1}{dt} &= \mathsf{k}^0_{\mathsf{ex}}\mathsf{MRN}_0 - \mathsf{k}^0_{\mathsf{im}}\mathsf{MRN}_1.
\end{aligned} \tag{3}$$

3 Modeling ATM Activation

We have introduced mechanistic models for the IR induced DSB and ROS production and chromatin relaxation, and chemical reaction model for the kinetics of MRN in the previous sections. As shown in Section 2.3, once a biological model is converted into a chemical reaction model, a mathematical model of the chemical reaction can be readily written in terms of differential equations by applying well known reaction laws such as law of mass action and Michealis-Menten kinetics. In the following, therefore, we will consider only how to build chemical reaction models in ATM activation. Their associated mathematical models will be provided at the end of the modeling session.

3.1 R1: Initiative ATM Activation and Signaling Chromatin Relaxation

In the model, we assume that ATM and Tip60 form a complex to be treated as an entity, denoted by TA, because they are always stably associated with each other. This complex is a monomer and may form a dimer dTA with another copy of itself. Usually ATM is bound with PP2A that inhibits ATM autophosphorylation, while Tip60 bound with ATF2 that inhibits the HAT activity of Tip60. Thus a TA dimer (dTA) bound with PP2A and ATF2 is the inactive form of ATM, denoted by dATAP.

It was proposed that ATM is activated due to the chromatin structure change [1], which hence is exclusively in the damaged site. Therefore, we assume that the rapid chromatin relaxation triggers the ATM activation by breaking inactive ATM dimer into active monomers and releasing $\mathsf{ATF2}$ and $\mathsf{PP2A}$ simultaneously. This may be caused by the instant collapse of compact structure of the heterochromatin and the release of the potential energy deposited during the DNA folding. Thus we think of the initiative ATM activation as a physical reaction. Such ATM activation may be in small amount because the relaxation is locally around the damage site, but this small amount of active ATM triggers a positive feedback loop to enhance ATM activation, as discussed in the main context and will be recalled shortly in the following sections. As mentioned in Section 2.2, ATM phosphorylates $\mathsf{KAP1}$ to have $\mathsf{KAP1}^{\mathsf{p}}$ that carries the signal of chromatin

relaxation. And $\mathsf{KAP1^p}$ may be dephosphorylated by some phosphatase to be $\mathsf{KAP1}$ that is assumed to carry the signal of chromatin condensation. Then the R1 reactions can be written as

$$\begin{array}{|ll|}\hline (1) & \mathsf{dATAP}_i \xrightarrow{\mathsf{k_{act}}\max\{\dot{\mathsf{r}}_i,0\}} 2\mathsf{TA}_i + 2\mathsf{PP2A}_i + 2\mathsf{ATF2}_i. \\ (2) & \mathsf{KAP1}_i \underset{\mathsf{k^1_{dp}}}{\overset{\mathsf{k^1_p aTA}_i}{\rightleftharpoons}} \mathsf{KAP1^p}_i, \\ \hline \end{array} \tag{R1}$$

where $\mathsf{k_{act}}$ is the efficiency rate of activation to the chromatin structure change, captured by $\dot{r}$, $\mathsf{k^1_p}$ the phosphorylation rate of $\mathsf{KAP1}$ by ATM and $\mathsf{k^1_{dp}}$ its dephosphorylation rate . Obviously $\dot{r} > 0$ means relaxation and $\dot{r} <$ means condensation. Note that reaction R1-1 is irreversible and $\dot{r} < 0$ has no contribution to ATM activation, thus $\dot{r}$ is replaced by $\max\{\dot{\mathsf{r}}_i, 0\}$.

In addition, as discussed in the main context, ATM is fully active in the form of monomer regardless of the presence of phosphorylation or acetylation, denoted by aTA; but only partially active if it is phosphorylated but remains a dimer, denoted by paTA. Then one has

$$\begin{aligned} \mathsf{aTA}_i &= (\mathsf{TA} + \mathsf{TA^a} + \mathsf{TA^p} + \mathsf{TA^{ap}})_i + (\mathsf{ATA} + \mathsf{ATA^p} + \mathsf{TAP} + \mathsf{TA^aP})_i + \mathsf{ATAP}_i \\ \mathsf{paTA}_i &= (\mathsf{dTA^p} + \mathsf{dATA^p})_i, \qquad\qquad i = 0, 1. \end{aligned}$$

Please refer to Table S1 for the interpretation of the notations. Because there is no evidence to show that $\mathsf{KAP1}$ is a substrate of partially active ATM, only aTA, but not paTA, is used in reaction R1-2.

3.2 R2: Tip60 Activation and ATM Acetylation

Reactions about the activation of Tip60 and acetylation of ATM are listed below.

$$\begin{array}{|llll|}\hline (1) & \mathsf{ATA}_i \xrightarrow{\mathsf{k^2_p aTA}_i} \mathsf{ATF2^p} + \mathsf{TA}_i, & (2) & \mathsf{dATA}_i \xrightarrow{\mathsf{k^2_p aTA}_i} 2\mathsf{ATF2^p} + \mathsf{dTA}_i \\ (3) & \mathsf{ATA^p_i} \xrightarrow{\mathsf{k^2_p aTA}_i} \mathsf{ATF2^p} + \mathsf{TA^p}_i, & (4) & \mathsf{dATA^p_i} \xrightarrow{\mathsf{k^2_p aTA}_i} 2\mathsf{ATF2^p} + \mathsf{dTA^p}_i \\ (5) & \mathsf{ATAP}_i \xrightarrow{\mathsf{k^2_p aTA}_i} \mathsf{ATF2^p} + \mathsf{TAP}_i, & (6) & \mathsf{dATAP}_i \xrightarrow{\mathsf{k^2_p aTA}_i} 2\mathsf{ATF2^p} + \mathsf{dTAP}_i \\ (7) & \mathsf{ATF2^p_i} \xrightarrow{\mathsf{k^2_{dp}}} \mathsf{ATF2}_i, & (8) & \mathsf{HKmHP1}_i \xrightarrow[\mathsf{r}_i]{\mathsf{k^3_p CK2}} \mathsf{H3K9me3}_i + \mathsf{HP1^p_i}, \\ (9) & \mathsf{HP1^p} \xrightarrow{\mathsf{k^3_{dp}}} \mathsf{HP1}, & (10) & \mathsf{H3K9me3} + \mathsf{HP1} \xrightarrow{\mathsf{k^6_a}} \mathsf{HKmHP1}, \\ (11) & \mathsf{TA}_i \xrightarrow[\mathsf{H3K9me3}]{\mathsf{k_{ace}r}_i} \mathsf{TA^a}_i, & (12) & \mathsf{dTA}_i \xrightarrow[\mathsf{H3K9me3}]{\mathsf{k_{ace}r}_i} \mathsf{dTA^a}_i, \\ (13) & \mathsf{TA^p_i} \xrightarrow[\mathsf{H3K9me3}]{\mathsf{k_{ace}r}_i} \mathsf{TA^{ap}}_i, & (14) & \mathsf{dTA^p_i} \xrightarrow[\mathsf{H3K9me3}]{\mathsf{k_{ace}r}_i} 2\mathsf{TA^{ap}}_i, \\ (15) & \mathsf{TAP}_i \xrightarrow[\mathsf{H3K9me3}]{\mathsf{k_{ace}r}_i} \mathsf{TA^aP}_i, & (16) & \mathsf{dTAP}_i \xrightarrow[\mathsf{H3K9me3}]{\mathsf{k_{ace}r}_i} \mathsf{dTA^aP}_i \\ \hline \end{array} \tag{R2}$$

$\mathsf{ATF2}$ is phosphorylated by active ATM and released from Tip60 at rate $\mathsf{k^2_p}$ (R2 (1-6)). On the other hand, in the relaxed chromatin (r dependent), $\mathsf{CK2}$ phosphorylates $\mathsf{HP1}$ at rate $\mathsf{k^3_p}$ so that $\mathsf{HP1}$ is released from $\mathsf{H3K9me3}$ and consequently

$\mathsf{H3K9me3}$ becomes exposed and accessible (R2-8). Without $\mathsf{ATF2}$, Tip60 can bind to $\mathsf{H3K9me3}$ in the relaxed chromatin to activate its HAT activity and acetylate ATM at rate $\mathsf{k_{ace}}$(R2 (11-16)). Different from reaction R2-14, reactions R2-12&16 show that acetylation on its own is not sufficient for initiating the dimer-monomer transition of the ATM protein, suggesting that additional events, such as autophosphorylation of ATM, may also be required [2]. Besides, we assume that $\mathsf{ATF2^p}$ (R2-7) and $\mathsf{HP1^p}$ (R2-9) are dephosphorylated by phosphatase, and free $\mathsf{HP1}$ may bind to $\mathsf{H3K9me3}$ (R2-10).

3.3 R3: Inhibition of PP2A by Oxidative Stress

It has been reported in [3], that under the IR-induced oxidative stress, $\mathsf{PP2A}$ dissociates from ATM (R3(1-6)) at rate $\mathsf{k_d^7}$ and translocates into caveolar membranes and interacts with caveolin-1 (R3-7).

$$\begin{array}{llll}
(1) & \mathsf{TAP}_i \xrightarrow{\mathsf{k_d^7 ROS}} \mathsf{TA}_i + \mathsf{PP2A}, & (2) & \mathsf{dTAP}_i \xrightarrow{\mathsf{k_d^7 ROS}} \mathsf{dTA}_i + 2\mathsf{PP2A}, \\
(3) & \mathsf{TA^aP}_i \xrightarrow{\mathsf{k_d^7 ROS}} \mathsf{TA^a}_i + \mathsf{PP2A}, & (4) & \mathsf{dTA^aP}_i \xrightarrow{\mathsf{k_d^7 ROS}} \mathsf{dTA^a}_i + 2\mathsf{PP2A}, \\
(5) & \mathsf{ATAP}_i \xrightarrow{\mathsf{k_d^7 ROS}} \mathsf{ATA}_i + \mathsf{PP2A}, & (6) & \mathsf{dATAP}_i \xrightarrow{\mathsf{k_d^7 ROS}} \mathsf{dATA}_i + 2\mathsf{PP2A},. \\
(7) & \mathsf{PP2A} + \mathsf{Cav} \underset{\mathsf{k_d^8}}{\overset{\mathsf{k_a^8 ROS}}{\rightleftharpoons}} \mathsf{CPP2A}. & &
\end{array} \tag{R3}$$

3.4 R4: Autophosphorylation of ATM

Once PP2A is released, ATM undergoes *in trans* autophosphorylation. ATM can be phosphorylated by either fully active ATM at rate $\mathsf{k_{ap}^1}$ or partially active ATM at rate $\mathsf{k_{ap}^2}$ (R4-1,3,5). If ATM is in the form of dimer, the autophosphorylation may be done between the associated binding pair at higher rate $\mathsf{k_{ap}^3}$ (R4-2,4,6). Furthermore, we assume Michaelis-Menten kinetics for the autophosphorylation of ATM monomers, and mass action kinetics for the autophosphorylation of ATM dimers without $\mathsf{PP2A}$ because of the presence of two ATM monomers close to each other.

$$\begin{array}{llll}
(1) & \mathsf{TA}_i \xrightarrow{\mathsf{k_{ap}^1 aTA}_i + \mathsf{k_{ap}^2 paTA}_i} \mathsf{TA^p}_i, & (2) & \mathsf{dTA}_i \xrightarrow{\mathsf{k_{ap}^3}} \mathsf{dTA^p}_i, \\
(3) & \mathsf{TA^a}_i \xrightarrow{\mathsf{k_{ap}^1 aTA}_i + \mathsf{k_{ap}^2 paTA}_i} \mathsf{TA^{ap}}_i, & (4) & \mathsf{dTA^a}_i \xrightarrow{\mathsf{k_{ap}^3}} 2\,\mathsf{TA^{ap}}_i, \\
(5) & \mathsf{ATA}_i \xrightarrow{\mathsf{k_{ap}^1 aTA}_i + \mathsf{k_{ap}^2 paTA}_i} \mathsf{ATA^p}_i, & (6) & \mathsf{dATA}_i \xrightarrow{\mathsf{k_{ap}^3}} \mathsf{dATA^p}_i.
\end{array} \tag{R4}$$

3.5 R5: Formation of High Order Complexes

Inactive ATM dimer dATAP can be formed through different pathways by changing the recruitment order of PP2A and ATF2 and formation of dimer. With all the possibilities of combination, the detailed reactions have been listed in R5.

(1)	$\mathrm{TA}_i + \mathrm{PP2A} \xrightarrow{k_a^1} \mathrm{TAP}_i,$	(2)	$\mathrm{TA^p}_i + \mathrm{PP2A} \xrightarrow{k_a^1} \mathrm{TAP}_i,$
(3)	$\mathrm{TA^a}_i + \mathrm{PP2A} \xrightarrow{k_a^1} \mathrm{TA^aP}_i,$	(4)	$\mathrm{TA^{ap}}_i + \mathrm{PP2A} \xrightarrow{k_a^1} \mathrm{TA^aP}_i,$
(5)	$\mathrm{ATF2} + \mathrm{TAP}_i \xrightarrow{k_a^2} \mathrm{ATAP}_i,$	(6)	$\mathrm{ATF2} + \mathrm{TA^aP}_i \xrightarrow{k_a^2} \mathrm{ATAP}_i,$
(7)	$\mathrm{ATF2} + \mathrm{TA}_i \xrightarrow{k_a^2} \mathrm{ATA}_i,$	(8)	$\mathrm{ATF2} + \mathrm{TA^a}_i \xrightarrow{k_a^2} \mathrm{ATA}_i,$
(9)	$\mathrm{ATF2} + \mathrm{TA^p}_i \xrightarrow{k_a^2} \mathrm{ATA^p}_i,$	(10)	$\mathrm{ATF2} + \mathrm{TA^{ap}}_i \xrightarrow{k_a^2} \mathrm{ATA^p}_i,$
(11)	$\mathrm{ATA}_i + \mathrm{PP2A} \xrightarrow{k_a^1} \mathrm{ATAP}_i,$	(12)	$\mathrm{ATA^p}_i + \mathrm{PP2A} \xrightarrow{k_a^1} \mathrm{ATAP}_i,$
(13)	$2\mathrm{TA}_i \xrightarrow{k_a^3} \mathrm{dTA},$	(14)	$2\mathrm{TA^a}_i \xrightarrow{k_a^3} \mathrm{dTA^a}$
(15)	$2\mathrm{TA^p}_i \xrightarrow{k_a^3} \mathrm{dTA^p},$	(16)	$2\mathrm{ATA}_i \xrightarrow{k_a^3} \mathrm{dATA},$
(17)	$2\mathrm{ATA^p}_i \xrightarrow{k_a^3} \mathrm{dATA^p},$	(18)	$2\mathrm{TAP}_i \xrightarrow{k_a^3} \mathrm{dTAP}$
(19)	$2\mathrm{TA^aP} \xrightarrow{k_a^3} \mathrm{dTA^aP}$	(20)	$2\mathrm{ATAP}_i \xrightarrow{k_a^3} \mathrm{dATAP}_i,$
(21)	$2\mathrm{ATF2} + \mathrm{dTA}_i \xrightarrow{k_a^4} \mathrm{dATA},$	(22)	$\mathrm{dTA}_i + 2\mathrm{PP2A} \xrightarrow{k_a^5} \mathrm{dTAP},$
(23)	$2\mathrm{ATF2} + \mathrm{dTA^a}_i \xrightarrow{k_a^4} \mathrm{dATA},$	(24)	$\mathrm{dTA^a}_i + 2\mathrm{PP2A} \xrightarrow{k_a^5} \mathrm{dTA^aP},$
(25)	$2\mathrm{ATF2} + \mathrm{dTA^p}_i \xrightarrow{k_a^4} \mathrm{dATA^p},$	(26)	$\mathrm{dTA^p}_i + 2\mathrm{PP2A} \xrightarrow{k_a^5} \mathrm{dTAP},$
(27)	$2\mathrm{ATF2} + \mathrm{dTAP}_i \xrightarrow{k_a^4} \mathrm{dATAP}_i,$	(28)	$\mathrm{dATA}_i + 2\mathrm{PP2A} \xrightarrow{k_a^5} \mathrm{dATAP}$
(29)	$2\mathrm{ATF2} + \mathrm{dTA^aP}_i \xrightarrow{k_a^4} \mathrm{dATAP}_i,$	(30)	$\mathrm{dATA^p}_i + 2\mathrm{PP2A} \xrightarrow{k_a^5} \mathrm{dATAP}.$

(R5)

3.6 R6: Shuttling between Damaged and Undamaged Sites

Finally, we consider most of the proteins that commute between the damaged site and undamaged site. The shuttling of any such protein can be written as a first order reversible chemical reaction with import rate k_{im} and export rate k_{ex}, see reaction R6. By taking into account the role of MRN to promote the recruitment of ATM to the DNA damage site, we assume the import and export

rates of ATM satisfy $\mathsf{k}^1_{\mathsf{im}} = \mathsf{k}^1_{\mathsf{ex}} + \mathsf{k}_{\mathsf{re}}\mathsf{DM}$, where k_{re} is the recruitment rate.

$$
\begin{array}{lll}
(1)\ \mathsf{TA}_0 \underset{\mathsf{k}^1_{\mathsf{im}}}{\overset{\mathsf{k}^1_{\mathsf{ex}}}{\rightleftharpoons}} \mathsf{TA}_1, &
(2)\ \mathsf{TA^a}_0 \underset{\mathsf{k}^1_{\mathsf{im}}}{\overset{\mathsf{k}^1_{\mathsf{ex}}}{\rightleftharpoons}} \mathsf{TA^a}_1, &
(3)\ \mathsf{TA^p}_0 \underset{\mathsf{k}^1_{\mathsf{im}}}{\overset{\mathsf{k}^1_{\mathsf{ex}}}{\rightleftharpoons}} \mathsf{TA^p}_1, \\
(4)\ \mathsf{TA^{ap}}_0 \underset{\mathsf{k}^1_{\mathsf{im}}}{\overset{\mathsf{k}^1_{\mathsf{ex}}}{\rightleftharpoons}} \mathsf{TA^{ap}}_1, &
(5)\ \mathsf{ATA}_0 \underset{\mathsf{k}^1_{\mathsf{im}}}{\overset{\mathsf{k}^1_{\mathsf{ex}}}{\rightleftharpoons}} \mathsf{ATA}_1, &
(6)\ \mathsf{ATA^p}_0 \underset{\mathsf{k}^1_{\mathsf{im}}}{\overset{\mathsf{k}^1_{\mathsf{ex}}}{\rightleftharpoons}} \mathsf{ATA^p}_1, \\
(7)\ \mathsf{TAP}_0 \underset{\mathsf{k}^1_{\mathsf{im}}}{\overset{\mathsf{k}^1_{\mathsf{ex}}}{\rightleftharpoons}} \mathsf{TAP}_1, &
(8)\ \mathsf{TA^aP}_0 \underset{\mathsf{k}^1_{\mathsf{im}}}{\overset{\mathsf{k}^1_{\mathsf{ex}}}{\rightleftharpoons}} \mathsf{TA^aP}_1, &
(9)\ \mathsf{ATAP}_0 \underset{\mathsf{k}^1_{\mathsf{im}}}{\overset{\mathsf{k}^1_{\mathsf{ex}}}{\rightleftharpoons}} \mathsf{ATAP}_1, \\
(10)\ \mathsf{dTA}_0 \underset{\mathsf{k}^1_{\mathsf{im}}}{\overset{\mathsf{k}^1_{\mathsf{ex}}}{\rightleftharpoons}} \mathsf{dTA}_1, &
(11)\ \mathsf{dTA^a}_0 \underset{\mathsf{k}^1_{\mathsf{im}}}{\overset{\mathsf{k}^1_{\mathsf{ex}}}{\rightleftharpoons}} \mathsf{dTA^a}_1, &
(12)\ \mathsf{dTA^p}_0 \underset{\mathsf{k}^1_{\mathsf{im}}}{\overset{\mathsf{k}^1_{\mathsf{ex}}}{\rightleftharpoons}} \mathsf{dTA^p}_1, \\
(13)\ \mathsf{dATA}_0 \underset{\mathsf{k}^1_{\mathsf{im}}}{\overset{\mathsf{k}^1_{\mathsf{ex}}}{\rightleftharpoons}} \mathsf{dATA}_1, &
(14)\ \mathsf{dATA^p}_0 \underset{\mathsf{k}^1_{\mathsf{im}}}{\overset{\mathsf{k}^1_{\mathsf{ex}}}{\rightleftharpoons}} \mathsf{dATA^p}_1, &
(15)\ \mathsf{dTAP}_0 \underset{\mathsf{k}^1_{\mathsf{im}}}{\overset{\mathsf{k}^1_{\mathsf{ex}}}{\rightleftharpoons}} \mathsf{dTAP}_1, \\
(16)\ \mathsf{dTA^aP}_0 \underset{\mathsf{k}^1_{\mathsf{im}}}{\overset{\mathsf{k}^1_{\mathsf{ex}}}{\rightleftharpoons}} \mathsf{dTA^aP}_1, &
(17)\ \mathsf{dATAP}_0 \underset{\mathsf{k}^1_{\mathsf{im}}}{\overset{\mathsf{k}^1_{\mathsf{ex}}}{\rightleftharpoons}} \mathsf{dATAP}_1, &
(18)\ \mathsf{KAP1}_0 \underset{\mathsf{k}^2_{\mathsf{im}}}{\overset{\mathsf{k}^2_{\mathsf{ex}}}{\rightleftharpoons}} \mathsf{KAP1}_1, \\
(19)\ \mathsf{KAP1}^{\mathsf{p}}_0 \underset{\mathsf{k}^2_{\mathsf{im}}}{\overset{\mathsf{k}^2_{\mathsf{ex}}}{\rightleftharpoons}} \mathsf{KAP1}^{\mathsf{p}}_1, &
(20)\ \mathsf{HP1}_0 \underset{\mathsf{k}^3_{\mathsf{im}}}{\overset{\mathsf{k}^3_{\mathsf{ex}}}{\rightleftharpoons}} \mathsf{HP1}_1, &
(21)\ \mathsf{HP1^p}_0 \underset{\mathsf{k}^3_{\mathsf{im}}}{\overset{\mathsf{k}^3_{\mathsf{ex}}}{\rightleftharpoons}} \mathsf{HP1^p}_1, \\
(22)\ \mathsf{ATF2}_0 \underset{\mathsf{k}^4_{\mathsf{im}}}{\overset{\mathsf{k}^4_{\mathsf{ex}}}{\rightleftharpoons}} \mathsf{ATF2}_1, &
(23)\ \mathsf{ATF2^p}_0 \underset{\mathsf{k}^4_{\mathsf{im}}}{\overset{\mathsf{k}^4_{\mathsf{ex}}}{\rightleftharpoons}} \mathsf{ATF2^p}_1, &
(24)\ \mathsf{PP2A}_0 \underset{\mathsf{k}^5_{\mathsf{im}}}{\overset{\mathsf{k}^5_{\mathsf{ex}}}{\rightleftharpoons}} \mathsf{PP2A}_1,
\end{array}
\tag{R6}
$$

4 Mathematical Model

4.1 Stoichiometric Matrix $\mathbb{S}$

The stoichiometric matrix of this reaction system is given by

$$
\mathbb{S} = \left[\begin{array}{c|c|c|c}
 & \mathbb{R}_0 & \mathbb{R}_1 & \mathbb{R}_{0-1} \\ \hline
\mathbb{X}_0 & A & 0 & -B \\ \hline
\mathbb{X}_1 & 0 & A & B
\end{array}\right], \tag{4}
$$

where

$$
A = \left[\begin{array}{c|c|c|c|c|c}
 & R_1 & R_2 & R_3 & R_4 & R_5 \\ \hline
X_1 & A_{11} & A_{12} & A_{13} & A_{14} & A_{15} \\ \hline
X_2 & A_{21} & A_{22} & A_{23} & A_{24} & A_{25} \\ \hline
X_3 & A_{31} & A_{32} & A_{33} & A_{34} & A_{35} \\ \hline
X_4 & A_{41} & A_{42} & A_{43} & A_{44} & A_{45} \\ \hline
X_5 & A_{51} & A_{52} & A_{53} & A_{54} & A_{55} \\ \hline
X_6 & A_{61} & A_{62} & A_{63} & A_{64} & A_{65}
\end{array}\right], \qquad
B = \left[\begin{array}{c|c}
 & R_6 \\ \hline
X_1 & B_1 \\ \hline
X_2 & B_2 \\ \hline
X_3 & B_3 \\ \hline
X_4 & B_4 \\ \hline
X_5 & B_5 \\ \hline
X_6 & B_6
\end{array}\right].
$$

Precisely

$$
A_{21} = 0_{5\times 2}, \qquad A_{31} = 0_{3\times 2}, \qquad A_{61} = 0_{4\times 2}
$$

$$
A_{11} = \begin{bmatrix} 2 & 0 \\ 0 & 0 \\ 0 & 0 \\ 0 & 0 \end{bmatrix}, \quad
A_{41} = \begin{bmatrix} 0 & 0 \\ 0 & 0 \\ 0 & 0 \\ 0 & 0 \\ -1 & 0 \end{bmatrix}, \quad
A_{51} = \begin{bmatrix} 0 & -1 \\ 0 & 1 \\ 0 & 0 \\ 0 & 0 \\ 2 & 0 \\ 0 & 0 \\ 2 & 0 \end{bmatrix},
$$

$$A_{12} = \begin{bmatrix} 1 & 0 & 0 & 0 & 0 & 0 & 0 & 0 & 0 & 0 & -1 & 0 & 0 & 0 & 0 & 0 \\ 0 & 0 & 0 & 0 & 0 & 0 & 0 & 0 & 0 & 0 & 1 & 0 & 0 & 0 & 0 & 0 \\ 0 & 0 & 1 & 0 & 0 & 0 & 0 & 0 & 0 & 0 & 0 & 0 & -1 & 0 & 0 & 0 \\ 0 & 0 & 0 & 0 & 0 & 0 & 0 & 0 & 0 & 0 & 0 & 0 & 1 & 2 & 0 & 0 \end{bmatrix}_{4\times16}$$

$$A_{22} = \begin{bmatrix} -1 & 0 & 0 & 0 & 0 & 0 & 0 & 0 & 0 & 0 & 0 & 0 & 0 & 0 & 0 & 0 \\ 0 & 0 & -1 & 0 & 0 & 0 & 0 & 0 & 0 & 0 & 0 & 0 & 0 & 0 & 0 & 0 \\ 0 & 0 & 0 & 0 & 1 & 0 & 0 & 0 & 0 & 0 & 0 & 0 & 0 & 0 & -1 & 0 \\ 0 & 0 & 0 & 0 & 0 & 0 & 0 & 0 & 0 & 0 & 0 & 0 & 0 & 0 & 1 & 0 \\ 0 & 0 & 0 & 0 & -1 & 0 & 0 & 0 & 0 & 0 & 0 & 0 & 0 & 0 & 0 & 0 \end{bmatrix}_{5\times16}$$

$$A_{32} = \begin{bmatrix} 0 & 1 & 0 & 0 & 0 & 0 & 0 & 0 & 0 & 0 & 0 & -1 & 0 & 0 & 0 & 0 \\ 0 & 0 & 0 & 0 & 0 & 0 & 0 & 0 & 0 & 0 & 0 & 1 & 0 & 0 & 0 & 0 \\ 0 & 0 & 0 & 1 & 0 & 0 & 0 & 0 & 0 & 0 & 0 & 0 & 0 & -1 & 0 & 0 \end{bmatrix}_{3\times16}$$

$$A_{42} = \begin{bmatrix} 0 & -1 & 0 & 0 & 0 & 0 & 0 & 0 & 0 & 0 & 0 & 0 & 0 & 0 & 0 & 0 \\ 0 & 0 & 0 & -1 & 0 & 0 & 0 & 0 & 0 & 0 & 0 & 0 & 0 & 0 & 0 & 0 \\ 0 & 0 & 0 & 0 & 0 & 1 & 0 & 0 & 0 & 0 & 0 & 0 & 0 & 0 & 0 & -1 \\ 0 & 0 & 0 & 0 & 0 & 0 & 0 & 0 & 0 & 0 & 0 & 0 & 0 & 0 & 0 & 1 \\ 0 & 0 & 0 & 0 & 0 & -1 & 0 & 0 & 0 & 0 & 0 & 0 & 0 & 0 & 0 & 0 \end{bmatrix}_{5\times16}$$

$$A_{52} = \begin{bmatrix} 0 & 0 & 0 & 0 & 0 & 0 & 0 & 0 & 0 & 0 & 0 & 0 & 0 & 0 & 0 & 0 \\ 0 & 0 & 0 & 0 & 0 & 0 & 0 & 0 & 0 & 0 & 0 & 0 & 0 & 0 & 0 & 0 \\ 0 & 0 & 0 & 0 & 0 & 0 & 0 & 0 & 1 & -1 & 0 & 0 & 0 & 0 & 0 & 0 \\ 0 & 0 & 0 & 0 & 0 & 0 & 0 & 1 & -1 & 0 & 0 & 0 & 0 & 0 & 0 & 0 \\ 0 & 0 & 0 & 0 & 0 & 0 & 1 & 0 & 0 & 0 & 0 & 0 & 0 & 0 & 0 & 0 \\ 1 & 2 & 1 & 2 & 1 & 2 & -1 & 0 & 0 & 0 & 0 & 0 & 0 & 0 & 0 & 0 \\ 0 & 0 & 0 & 0 & 0 & 0 & 0 & 0 & 0 & 0 & 0 & 0 & 0 & 0 & 0 & 0 \end{bmatrix}_{8\times16}$$

$$A_{62} = \begin{bmatrix} 0 & 0 & 0 & 0 & 0 & 0 & 0 & 1 & 0 & -1 & 0 & 0 & 0 & 0 & 0 & 0 \\ 0 & 0 & 0 & 0 & 0 & 0 & 0 & -1 & 0 & 1 & 0 & 0 & 0 & 0 & 0 & 0 \\ 0 & 0 & 0 & 0 & 0 & 0 & 0 & 0 & 0 & 0 & 0 & 0 & 0 & 0 & 0 & 0 \\ 0 & 0 & 0 & 0 & 0 & 0 & 0 & 0 & 0 & 0 & 0 & 0 & 0 & 0 & 0 & 0 \end{bmatrix}_{8\times16}$$

$$A_{13} = \begin{bmatrix} 1 & 0 & 0 & 0 & 0 & 0 & 0 \\ 0 & 0 & 1 & 0 & 0 & 0 & 0 \\ 0 & 0 & 0 & 0 & 0 & 0 & 0 \\ 0 & 0 & 0 & 0 & 0 & 0 & 0 \end{bmatrix}_{4\times7}$$

$$A_{33} = \begin{bmatrix} 0 & 1 & 0 & 0 & 0 & 0 & 0 \\ 0 & 0 & 0 & 1 & 0 & 0 & 0 \\ 0 & 0 & 0 & 0 & 0 & 0 & 0 \end{bmatrix}_{3\times7}$$

$$A_{53} = \begin{bmatrix} 0 & 0 & 0 & 0 & 0 & 0 & 0 \\ 0 & 0 & 0 & 0 & 0 & 0 & 0 \\ 0 & 0 & 0 & 0 & 0 & 0 & 0 \\ 0 & 0 & 0 & 0 & 0 & 0 & 0 \\ 0 & 0 & 0 & 0 & 0 & 0 & 0 \\ 0 & 0 & 0 & 0 & 0 & 0 & 0 \\ 1 & 2 & 1 & 2 & 1 & 2 & -1 \end{bmatrix}_{7\times7}$$

$$A_{23} = \begin{bmatrix} 0 & 0 & 0 & 0 & 1 & 0 & 0 \\ 0 & 0 & 0 & 0 & 0 & 0 & 0 \\ -1 & 0 & 0 & 0 & 0 & 0 & 0 \\ 0 & 0 & -1 & 0 & 0 & 0 & 0 \\ 0 & 0 & 0 & 0 & -1 & 0 & 0 \end{bmatrix}_{5\times7}$$

$$A_{43} = \begin{bmatrix} 0 & 0 & 0 & 0 & 0 & 1 & 0 \\ 0 & 0 & 0 & 0 & 0 & 0 & 0 \\ 0 & -1 & 0 & 0 & 0 & 0 & 0 \\ 0 & 0 & 0 & -1 & 0 & 0 & 0 \\ 0 & 0 & 0 & 0 & 0 & -1 & 0 \end{bmatrix}_{5\times7}$$

$$A_{63} = \begin{bmatrix} 0 & 0 & 0 & 0 & 0 & 0 & 0 \\ 0 & 0 & 0 & 0 & 0 & 0 & 0 \\ 0 & 0 & 0 & 0 & 0 & 0 & -1 \\ 0 & 0 & 0 & 0 & 0 & 0 & 1 \end{bmatrix}_{4\times7}$$

$$A_{14} = \begin{bmatrix} -1 & 0 & 0 & 0 & 0 & 0 \\ 0 & 0 & -1 & 0 & 0 & 0 \\ 1 & 0 & 0 & 0 & 0 & 0 \\ 0 & 0 & 1 & 2 & 0 & 0 \end{bmatrix}_{4\times6}$$

$$A_{34} = \begin{bmatrix} 0 & -1 & 0 & 0 & 0 & 0 \\ 0 & 0 & 0 & -1 & 0 & 0 \\ 0 & 1 & 0 & 0 & 0 & 0 \end{bmatrix}_{3\times6}$$

$$A_{54} = 0_{7\times6}$$

$$A_{24} = \begin{bmatrix} 0 & 0 & 0 & 0 & -1 & 0 \\ 0 & 0 & 0 & 0 & 1 & 0 \\ 0 & 0 & 0 & 0 & 0 & 0 \\ 0 & 0 & 0 & 0 & 0 & 0 \\ 0 & 0 & 0 & 0 & 0 & 0 \end{bmatrix}_{5\times6}$$

$$A_{44} = \begin{bmatrix} 0 & 0 & 0 & 0 & 0 & -1 \\ 0 & 0 & 0 & 0 & 0 & 1 \\ 0 & 0 & 0 & 0 & 0 & 0 \\ 0 & 0 & 0 & 0 & 0 & 0 \\ 0 & 0 & 0 & 0 & 0 & 0 \end{bmatrix}_{5\times6}$$

$$A_{64} = 0_{4\times6}$$

In addtion,

$$B = \begin{bmatrix} I_{24\times24} \\ 0_{4\times24} \end{bmatrix}$$

Therefore

$$A = \left[\begin{array}{c|c|c|c|c|c} & R_1 & R_2 & R_3 & R_4 & R_5 \\ \hline X_1 & A_{11} & A_{12} & A_{13} & A_{14} & A_{15} \\ \hline X_2 & 0 & A_{22} & A_{23} & A_{24} & A_{25} \\ \hline X_3 & 0 & A_{32} & A_{33} & A_{34} & A_{35} \\ \hline X_4 & A_{41} & A_{42} & A_{43} & A_{44} & A_{45} \\ \hline X_5 & A_{51} & A_{52} & A_{53} & 0 & A_{55} \\ \hline X_6 & 0 & A_{62} & A_{63} & 0 & 0 \end{array}\right], \qquad B = \left[\begin{array}{c|c} & R_6 \\ \hline X_1 & B_1 \\ \hline X_2 & B_2 \\ \hline X_3 & B_3 \\ \hline X_4 & B_4 \\ \hline X_5 & B_5 \\ \hline X_6 & B_6 \end{array}\right].$$

$$A_{15} = \left[\begin{array}{cccccccccccccccccccccccccccccc}
1 & 2 & 3 & 4 & 5 & 6 & 7 & 8 & 9 & 10 & 11 & 12 & 13 & 14 & 15 & 16 & 17 & 18 & 19 & 20 & 21 & 22 & 23 & 24 & 25 & 26 & 27 & 28 & 29 & 30 \\ \hline
-1 & 0 & 0 & 0 & 0 & 0 & -1 & 0 & 0 & 0 & 0 & 0 & -2 & 0 & 0 & 0 & 0 & 0 & 0 & 0 & 0 & 0 & 0 & 0 & 0 & 0 & 0 & 0 & 0 & 0 \\
0 & 0 & -1 & 0 & 0 & 0 & 0 & -1 & 0 & 0 & 0 & 0 & 0 & -2 & 0 & 0 & 0 & 0 & 0 & 0 & 0 & 0 & 0 & 0 & 0 & 0 & 0 & 0 & 0 & 0 \\
0 & -1 & 0 & 0 & 0 & 0 & 0 & 0 & -1 & 0 & 0 & 0 & 0 & 0 & -2 & 0 & 0 & 0 & 0 & 0 & 0 & 0 & 0 & 0 & 0 & 0 & 0 & 0 & 0 & 0 \\
0 & 0 & 0 & -1 & 0 & 0 & 0 & 0 & 0 & -1 & 0 & 0 & 0 & 0 & 0 & 0 & 0 & 0 & 0 & 0 & 0 & 0 & 0 & 0 & 0 & 0 & 0 & 0 & 0 & 0
\end{array}\right]_{4\times 30}$$

$$A_{25} = \left[\begin{array}{cccccccccccccccccccccccccccccc}
1 & 2 & 3 & 4 & 5 & 6 & 7 & 8 & 9 & 10 & 11 & 12 & 13 & 14 & 15 & 16 & 17 & 18 & 19 & 20 & 21 & 22 & 23 & 24 & 25 & 26 & 27 & 28 & 29 & 30 \\ \hline
0 & 0 & 0 & 0 & 0 & 0 & 1 & 1 & 0 & 0 & -1 & 0 & 0 & 0 & 0 & -2 & 0 & 0 & 0 & 0 & 0 & 0 & 0 & 0 & 0 & 0 & 0 & 0 & 0 & 0 \\
0 & 0 & 0 & 0 & 0 & 0 & 0 & 0 & 1 & 1 & 0 & -1 & 0 & 0 & 0 & 0 & -2 & 0 & 0 & 0 & 0 & 0 & 0 & 0 & 0 & 0 & 0 & 0 & 0 & 0 \\
1 & 1 & 0 & 0 & -1 & 0 & 0 & 0 & 0 & 0 & 0 & 0 & 0 & 0 & 0 & 0 & 0 & -2 & 0 & 0 & 0 & 0 & 0 & 0 & 0 & 0 & 0 & 0 & 0 & 0 \\
0 & 0 & 1 & 1 & 0 & -1 & 0 & 0 & 0 & 0 & 0 & 0 & 0 & 0 & 0 & 0 & 0 & 0 & -2 & 0 & 0 & 0 & 0 & 0 & 0 & 0 & 0 & 0 & 0 & 0 \\
0 & 0 & 0 & 0 & 1 & 1 & 0 & 0 & 0 & 0 & 1 & 1 & 0 & 0 & 0 & 0 & 0 & 0 & 0 & -2 & 0 & 0 & 0 & 0 & 0 & 0 & 0 & 0 & 0 & 0
\end{array}\right]_{5\times 30}$$

$$A_{35} = \left[\begin{array}{cccccccccccccccccccccccccccccc}
1 & 2 & 3 & 4 & 5 & 6 & 7 & 8 & 9 & 10 & 11 & 12 & 13 & 14 & 15 & 16 & 17 & 18 & 19 & 20 & 21 & 22 & 23 & 24 & 25 & 26 & 27 & 28 & 29 & 30 \\ \hline
0 & 0 & 0 & 0 & 0 & 0 & 0 & 0 & 0 & 0 & 0 & 0 & 1 & 0 & 0 & 0 & 0 & 0 & 0 & 0 & -1 & -1 & 0 & 0 & 0 & 0 & 0 & 0 & 0 & 0 \\
0 & 0 & 0 & 0 & 0 & 0 & 0 & 0 & 0 & 0 & 0 & 0 & 0 & 1 & 0 & 0 & 0 & 0 & 0 & 0 & 0 & 0 & -1 & -1 & 0 & 0 & 0 & 0 & 0 & 0 \\
0 & 0 & 0 & 0 & 0 & 0 & 0 & 0 & 0 & 0 & 0 & 0 & 0 & 0 & 1 & 0 & 0 & 0 & 0 & 0 & 0 & 0 & 0 & 0 & -1 & -1 & 0 & 0 & 0 & 0
\end{array}\right]_{3\times 30}$$

$$A_{45} = \left[\begin{array}{cccccccccccccccccccccccccccccc}
1 & 2 & 3 & 4 & 5 & 6 & 7 & 8 & 9 & 10 & 11 & 12 & 13 & 14 & 15 & 16 & 17 & 18 & 19 & 20 & 21 & 22 & 23 & 24 & 25 & 26 & 27 & 28 & 29 & 30 \\ \hline
0 & 0 & 0 & 0 & 0 & 0 & 0 & 0 & 0 & 0 & 0 & 0 & 0 & 0 & 0 & 1 & 0 & 0 & 0 & 0 & 1 & 0 & 1 & 0 & 0 & 0 & 0 & -1 & 0 & 0 \\
0 & 0 & 0 & 0 & 0 & 0 & 0 & 0 & 0 & 0 & 0 & 0 & 0 & 0 & 0 & 0 & 1 & 0 & 0 & 0 & 0 & 0 & 0 & 0 & 1 & 0 & 0 & 0 & 0 & -1 \\
0 & 0 & 0 & 0 & 0 & 0 & 0 & 0 & 0 & 0 & 0 & 0 & 0 & 0 & 0 & 0 & 0 & 1 & 0 & 0 & 0 & 1 & 0 & 0 & 0 & 1 & -1 & 0 & 0 & 0 \\
0 & 0 & 0 & 0 & 0 & 0 & 0 & 0 & 0 & 0 & 0 & 0 & 0 & 0 & 0 & 0 & 0 & 0 & 1 & 0 & 0 & 0 & 0 & 1 & 0 & 0 & 0 & 0 & -1 & 0 \\
0 & 0 & 0 & 0 & 0 & 0 & 0 & 0 & 0 & 0 & 0 & 0 & 0 & 0 & 0 & 0 & 0 & 0 & 0 & 1 & 0 & 0 & 0 & 0 & 0 & 0 & 1 & 1 & 1 & 1
\end{array}\right]_{5\times 30}$$

$$A_{55} = \left[\begin{array}{cccccccccccccccccccccccccccccc}
1 & 2 & 3 & 4 & 5 & 6 & 7 & 8 & 9 & 10 & 11 & 12 & 13 & 14 & 15 & 16 & 17 & 18 & 19 & 20 & 21 & 22 & 23 & 24 & 25 & 26 & 27 & 28 & 29 & 30 \\ \hline
0 & 0 & 0 & 0 & 0 & 0 & 0 & 0 & 0 & 0 & 0 & 0 & 0 & 0 & 0 & 0 & 0 & 0 & 0 & 0 & 0 & 0 & 0 & 0 & 0 & 0 & 0 & 0 & 0 & 0 \\
0 & 0 & 0 & 0 & 0 & 0 & 0 & 0 & 0 & 0 & 0 & 0 & 0 & 0 & 0 & 0 & 0 & 0 & 0 & 0 & 0 & 0 & 0 & 0 & 0 & 0 & 0 & 0 & 0 & 0 \\
0 & 0 & 0 & 0 & 0 & 0 & 0 & 0 & 0 & 0 & 0 & 0 & 0 & 0 & 0 & 0 & 0 & 0 & 0 & 0 & 0 & 0 & 0 & 0 & 0 & 0 & 0 & 0 & 0 & 0 \\
0 & 0 & 0 & 0 & 0 & 0 & 0 & 0 & 0 & 0 & 0 & 0 & 0 & 0 & 0 & 0 & 0 & 0 & 0 & 0 & 0 & 0 & 0 & 0 & 0 & 0 & 0 & 0 & 0 & 0 \\
0 & 0 & 0 & 0 & -1 & -1 & -1 & -1 & -1 & -1 & 0 & 0 & 0 & 0 & 0 & 0 & 0 & 0 & 0 & 0 & -2 & 0 & -2 & 0 & -2 & 0 & -2 & 0 & -2 & 0 \\
0 & 0 & 0 & 0 & 0 & 0 & 0 & 0 & 0 & 0 & 0 & 0 & 0 & 0 & 0 & 0 & 0 & 0 & 0 & 0 & 0 & 0 & 0 & 0 & 0 & 0 & 0 & 0 & 0 & 0 \\
-1 & -1 & -1 & -1 & 0 & 0 & 0 & 0 & 0 & 0 & -1 & -1 & 0 & 0 & 0 & 0 & 0 & 0 & 0 & 0 & 0 & -2 & 0 & -2 & 0 & -2 & 0 & -2 & 0 & -2
\end{array}\right]_{7\times 30}$$

$$A_{65} = 0_{4\times 30}$$

4.2 Reaction Fluxes $\mathbb{R}$

$R_1^i(1)$	=	$\mathrm{k_{act}}\dot{\mathrm{r}}_i\mathrm{dATAP}_i$	$R_1^i(2)$	=	$\dfrac{\mathrm{k}_\mathrm{p}^1\mathrm{aTA}_i\mathrm{KAP1}_i}{\mathrm{K}_1+\mathrm{KAP1}_i}-\dfrac{\mathrm{k}_\mathrm{dp}^1\mathrm{KAP1}^\mathrm{p}_i}{\mathrm{K}_1+\mathrm{KAP1^p}_i}$
$R_2^i(1)$	=	$\dfrac{\mathrm{k}_\mathrm{p}^2\mathrm{aTA}_i\mathrm{ATA}_i}{\mathrm{K}_2+\mathrm{ATA}_i}$	$R_2^i(2)$	=	$\dfrac{\mathrm{k}_\mathrm{p}^2\mathrm{aTA}_i\mathrm{dATA}_i}{\mathrm{K}_2+\mathrm{dATA}_i}$
$R_2^i(3)$	=	$\dfrac{\mathrm{k}_\mathrm{p}^2\mathrm{aTA}_i\mathrm{ATA^p}_i}{\mathrm{K}_2+\mathrm{ATA^p}_i}$	$R_2^i(4)$	=	$\dfrac{\mathrm{k}_\mathrm{p}^2\mathrm{aTA}_i\mathrm{dATA^p}_i}{\mathrm{K}_2+\mathrm{dATA^p}_i}$
$R_2^i(5)$	=	$\dfrac{\mathrm{k}_\mathrm{p}^2\mathrm{aTA}_i\mathrm{ATAP}_i}{\mathrm{K}_2+\mathrm{ATAP}_i}$	$R_2^i(6)$	=	$\dfrac{\mathrm{k}_\mathrm{p}^2\mathrm{aTA}_i\mathrm{dATAP}_i}{\mathrm{K}_2+\mathrm{dATAP}_i}$
$R_2^i(7)$	=	$\dfrac{\mathrm{k}_\mathrm{dp}^2\mathrm{ATF2^p}_i}{\mathrm{K}_2+\mathrm{ATF2^p}_i}$	$R_2^i(8)$	=	$\dfrac{\mathrm{k}_\mathrm{p}^3\mathrm{r}_i\mathrm{CK2\,HKmHP1}_i}{\mathrm{K}_3+\mathrm{HKmHP1}_i}$
$R_2^i(9)$	=	$\dfrac{\mathrm{k}_\mathrm{dp}^3\mathrm{HP1^p}_i}{\mathrm{K}_3+\mathrm{HP1^p}_i}$	$R_2^i(10)$	=	$\mathrm{k}_\mathrm{a}^6\mathrm{H3K9me3}_i\mathrm{HP1}_i$
$R_2^i(11)$	=	$\mathrm{k_{ace}H3K9me3}_i\mathrm{TA}_i$	$R_2^i(12)$	=	$\mathrm{k_{ace}H3K9me3}_i\mathrm{dTA}_i$
$R_2^i(13)$	=	$\mathrm{k_{ace}H3K9me3}_i\mathrm{TA^p}_i$	$R_2^i(14)$	=	$\mathrm{k_{ace}H3K9me3}_i\mathrm{dTA^p}_i$
$R_2^i(15)$	=	$\mathrm{k_{ace}H3K9me3}_i\mathrm{TAP}_i$	$R_2^i(16)$	=	$\mathrm{k_{ace}H3K9me3}_i\mathrm{dTAP}_i$
$R_3^i(1)$	=	$\mathrm{k}_\mathrm{d}^7\mathrm{ROS}_i\mathrm{TAP}_i$	$R_3^i(2)$	=	$\mathrm{k}_\mathrm{d}^7\mathrm{ROS}_i\mathrm{dTAP}_i$
$R_3^i(3)$	=	$\mathrm{k}_\mathrm{d}^7\mathrm{ROS}_i\mathrm{TA^aP}_i$	$R_3^i(4)$	=	$\mathrm{k}_\mathrm{d}^7\mathrm{ROS}_i\mathrm{dTA^aP}_i$
$R_3^i(5)$	=	$\mathrm{k}_\mathrm{d}^7\mathrm{ROS}_i\mathrm{ATAP}_i$	$R_3^i(6)$	=	$\mathrm{k}_\mathrm{d}^7\mathrm{ROS}_i\mathrm{dATAP}_i$
$R_3^i(7)$	=	$\mathrm{k}_\mathrm{a}^8\mathrm{ROS}_i\mathrm{Cav}_i\mathrm{PP2A}_i-\mathrm{k}_\mathrm{d}^8\mathrm{CPP2A}_i$			
$R_4^i(1)$	=	$\left(\frac{\mathrm{k}_\mathrm{ap}^1\mathrm{aTA}_i}{\mathrm{K}_4+\mathrm{TA}_i}+\frac{\mathrm{k}_\mathrm{ap}^2\mathrm{paTA}_i}{\mathrm{K}_5+\mathrm{TA}_i}\right)\mathrm{TA}_i$	$R_4^i(2)$	=	$\mathrm{k}_\mathrm{ap}^3\mathrm{dTA}_i$
$R_4^i(3)$	=	$\left(\frac{\mathrm{k}_\mathrm{ap}^1\mathrm{aTA}_i}{\mathrm{K}_4+\mathrm{TA^a}_i}+\frac{\mathrm{k}_\mathrm{ap}^2\mathrm{paTA}_i}{\mathrm{K}_5+\mathrm{TA^a}_i}\right)\mathrm{TA^a}_i$	$R_4^i(4)$	=	$\mathrm{k}_\mathrm{ap}^3\mathrm{dTA^a}_i$
$R_4^i(5)$	=	$\left(\frac{\mathrm{k}_\mathrm{ap}^1\mathrm{aTA}_i}{\mathrm{K}_4+\mathrm{ATA}_i}+\frac{\mathrm{k}_\mathrm{ap}^2\mathrm{paTA}_i}{\mathrm{K}_5+\mathrm{ATA}_i}\right)\mathrm{ATA}_i$	$R_4^i(6)$	=	$\mathrm{k}_\mathrm{ap}^3\mathrm{dATA}_i$
$R_5^i(1)$	=	$\mathrm{k}_\mathrm{a}^1\mathrm{PP2A}_i\mathrm{TA}_i$	$R_5^i(2)$	=	$\mathrm{k}_\mathrm{a}^1\mathrm{PP2A}_i\mathrm{TA^p}_i$
$R_5^i(3)$	=	$\mathrm{k}_\mathrm{a}^1\mathrm{PP2A}_i\mathrm{TA^a}_i$	$R_5^i(4)$	=	$\mathrm{k}_\mathrm{a}^1\mathrm{PP2A}_i\mathrm{TA^{ap}}_i$
$R_5^i(5)$	=	$\mathrm{k}_\mathrm{a}^2\mathrm{ATF2}_i\mathrm{TAP}_i$	$R_5^i(6)$	=	$\mathrm{k}_\mathrm{a}^2\mathrm{ATF2}_i\mathrm{TA^aP}_i$
$R_5^i(7)$	=	$\mathrm{k}_\mathrm{a}^2\mathrm{ATF2}_i\mathrm{TA}_i$	$R_5^i(8)$	=	$\mathrm{k}_\mathrm{a}^2\mathrm{ATF2}_i\mathrm{TA^a}_i$
$R_5^i(9)$	=	$\mathrm{k}_\mathrm{a}^2\mathrm{ATF2}_i\mathrm{TA^p}_i$	$R_5^i(10)$	=	$\mathrm{k}_\mathrm{a}^2\mathrm{ATF2}_i\mathrm{TA^{ap}}_i$
$R_5^i(11)$	=	$\mathrm{k}_\mathrm{a}^1\mathrm{PP2A}_i\mathrm{ATA}_i$	$R_5^i(12)$	=	$\mathrm{k}_\mathrm{a}^1\mathrm{PP2A}_i\mathrm{ATA^p}_i$
$R_5^i(13)$	=	$\mathrm{k}_\mathrm{a}^3\mathrm{TA}_i^2,$	$R_5^i(14)$	=	$\mathrm{k}_\mathrm{a}^3\mathrm{TA^a}{}_i^2$
$R_5^i(15)$	=	$\mathrm{k}_\mathrm{a}^3\mathrm{TA^p}{}_i^2,$	$R_5^i(16)$	=	$\mathrm{k}_\mathrm{a}^3\mathrm{ATA}_i^2$
$R_5^i(17)$	=	$\mathrm{k}_\mathrm{a}^3\mathrm{ATA^p}{}_i^2,$	$R_5^i(18)$	=	$\mathrm{k}_\mathrm{a}^3\mathrm{TAP}_i^2$
$R_5^i(19)$	=	$\mathrm{k}_\mathrm{a}^3\mathrm{TA^aP}_i^2,$	$R_5^i(20)$	=	$\mathrm{k}_\mathrm{a}^3\mathrm{ATAP}_i^2$
$R_5^i(21)$	=	$\mathrm{k}_\mathrm{a}^4\mathrm{ATF2}_i^2\mathrm{dTA}_i$	$R_5^i(22)$	=	$\mathrm{k}_\mathrm{a}^5\mathrm{PP2A}_i^2\mathrm{dTA}_i$
$R_5^i(23)$	=	$\mathrm{k}_\mathrm{a}^4\mathrm{ATF2}_i^2\mathrm{dTA^a}_i$	$R_5^i(24)$	=	$\mathrm{k}_\mathrm{a}^5\mathrm{PP2A}_i^2\mathrm{dTA^a}_i$
$R_5^i(25)$	=	$\mathrm{k}_\mathrm{a}^4\mathrm{ATF2}_i^2\mathrm{dTA^p}_i$	$R_5^i(26)$	=	$\mathrm{k}_\mathrm{a}^5\mathrm{PP2A}_i^2\mathrm{dTA^p}_i$
$R_5^i(27)$	=	$\mathrm{k}_\mathrm{a}^4\mathrm{ATF2}_i^2\mathrm{dTAP}_i$	$R_5^i(28)$	=	$\mathrm{k}_\mathrm{a}^5\mathrm{PP2A}_i^2\mathrm{dATA}_i$
$R_5^i(29)$	=	$\mathrm{k}_\mathrm{a}^4\mathrm{ATF2}_i^2\mathrm{dTA^aP}_i$	$R_5^i(30)$	=	$\mathrm{k}_\mathrm{a}^5\mathrm{PP2A}_i^2\mathrm{dATA^p}_i$
$R_6(1)$	=	$\mathrm{k}_\mathrm{ex}^1\mathrm{TA}_0-\mathrm{k}_\mathrm{im}^1\mathrm{TA}_1$	$R_6(2)$	=	$\mathrm{k}_\mathrm{ex}^1\mathrm{TA^a}_0-\mathrm{k}_\mathrm{im}^1\mathrm{TA^a}_1$
$R_6(3)$	=	$\mathrm{k}_\mathrm{ex}^1\mathrm{TA^p}_0-\mathrm{k}_\mathrm{im}^1\mathrm{TA^p}_1$	$R_6(4)$	=	$\mathrm{k}_\mathrm{ex}^1\mathrm{TA^{ap}}_0-\mathrm{k}_\mathrm{im}^1\mathrm{TA^{ap}}_1$
$R_6(5)$	=	$\mathrm{k}_\mathrm{ex}^1\mathrm{ATA}_0-\mathrm{k}_\mathrm{im}^1\mathrm{ATA}_1$	$R_6(6)$	=	$\mathrm{k}_\mathrm{ex}^1\mathrm{ATA^p}_0-\mathrm{k}_\mathrm{im}^1\mathrm{ATA^p}_1$
$R_6(7)$	=	$\mathrm{k}_\mathrm{ex}^1\mathrm{TAP}_0-\mathrm{k}_\mathrm{im}^1\mathrm{TAP}_1$	$R_6(8)$	=	$\mathrm{k}_\mathrm{ex}^1\mathrm{TA^aP}_0-\mathrm{k}_\mathrm{im}^1\mathrm{TA^aP}_1$
$R_6(9)$	=	$\mathrm{k}_\mathrm{ex}^1\mathrm{ATAP}_0-\mathrm{k}_\mathrm{im}^1\mathrm{ATAP}_1$	$R_6(10)$	=	$\mathrm{k}_\mathrm{ex}^1\mathrm{dTA}_0-\mathrm{k}_\mathrm{im}^1\mathrm{dTA}_1$
$R_6(11)$	=	$\mathrm{k}_\mathrm{ex}^1\mathrm{dTA^a}_0-\mathrm{k}_\mathrm{im}^1\mathrm{dTA^a}_1$	$R_6(12)$	=	$\mathrm{k}_\mathrm{ex}^1\mathrm{dTA^p}_0-\mathrm{k}_\mathrm{im}^1\mathrm{dTA^p}_1$
$R_6(13)$	=	$\mathrm{k}_\mathrm{ex}^1\mathrm{dATA}_0-\mathrm{k}_\mathrm{im}^1\mathrm{dATA}_1$	$R_6(14)$	=	$\mathrm{k}_\mathrm{ex}^1\mathrm{dATA^p}_0-\mathrm{k}_\mathrm{im}^1\mathrm{dATA^p}_1$
$R_6(15)$	=	$\mathrm{k}_\mathrm{ex}^1\mathrm{dTAP}_0-\mathrm{k}_\mathrm{im}^1\mathrm{dTAP}_1$	$R_6(16)$	=	$\mathrm{k}_\mathrm{ex}^1\mathrm{dTA^aP}_0-\mathrm{k}_\mathrm{im}^1\mathrm{dTA^aP}_1$
$R_6(17)$	=	$\mathrm{k}_\mathrm{ex}^1\mathrm{dATAP}_0-\mathrm{k}_\mathrm{im}^1\mathrm{dATAP}_1$	$R_6(18)$	=	$\mathrm{k}_\mathrm{ex}^2\mathrm{KAP1}_0-\mathrm{k}_\mathrm{im}^2\mathrm{KAP1}_1$
$R_6(19)$	=	$\mathrm{k}_\mathrm{ex}^2\mathrm{KAP1^p}_0-\mathrm{k}_\mathrm{im}^2\mathrm{KAP1^p}_1$	$R_6(20)$	=	$\mathrm{k}_\mathrm{ex}^3\mathrm{HP1}_0-\mathrm{k}_\mathrm{im}^3\mathrm{HP1}_1$
$R_6(21)$	=	$\mathrm{k}_\mathrm{ex}^3\mathrm{HP1^p}_0-\mathrm{k}_\mathrm{im}^3\mathrm{HP1^p}_1$	$R_6(22)$	=	$\mathrm{k}_\mathrm{ex}^4\mathrm{ATF2}_0-\mathrm{k}_\mathrm{im}^4\mathrm{ATF2}_1$
$R_6(23)$	=	$\mathrm{k}_\mathrm{ex}^4\mathrm{ATF2^p}_0-\mathrm{k}_\mathrm{im}^4\mathrm{ATF2^p}_1$	$R_6(24)$	=	$\mathrm{k}_\mathrm{ex}^5\mathrm{PP2A}_0-\mathrm{k}_\mathrm{im}^5\mathrm{PP2A}_1$

4.3 Reaction Rate Equation

The reaction rate equation is given by

$$\dot{\mathbb{X}} = \mathbb{S}\mathbb{R}(\mathbb{X}). \tag{5}$$

Along with equations (1-3), equation (5) forms a complete mathematical model for the ATM activation, that describes the dynamical change of the participating species.

5 Parameters

In the model, we assume acute dose that is given by

$$\mathsf{D_R}(t) = \begin{cases} \mathsf{r_D}, & t \in [0, \mathsf{t_D}], \\ 0, & t > \mathsf{t_D}, \end{cases}$$

where $\mathsf{r_D}$ is the radiation dose rate and $\mathsf{t_D}$ is the time duration in which the radiation dose is given. In addition, we assume that the relaxation rate changes induced by DSB and by $\mathsf{KAP1^P}$ are given by

$$f(x) = \frac{x}{\mathsf{K_r^1} + x}, \qquad g(x) = \frac{x}{\mathsf{K_r^2} + x}.$$

respectively. The condensation rate change related to $\mathsf{KAP1}$ is given by

$$h(x) = \frac{x}{\mathsf{K_r^3} + x}.$$

The values of the parameters are hard to meansure experimentally. In the following, we assume that all the parameters are dimensionless. The numerical simulation is conducted with the following estimates of all the involving parameters.

Parameter	Value	Parameter	Value	Parameter	Value
t_D	0.5	r_D	1	$CK2$	1
b_{DSB}	0.5	r_{DSB}	1		
b_{ROS}	0.5	d_{ROS}	1	r_{ROS}	1
b_{DR}	0.5	b_{RD}	1		
K_r^1	1	K_r^2	1	K_r^3	1
k_{act}	1	k_{re}	5		
k_p^1	1	k_{dp}^1	2		
k_p^2	1	k_{dp}^2	1		
k_p^3	1	k_{dp}^3	1		
K_1	0.2	K_2	0.2	K_3	0.3
K_4	0.3	K_5	0.5		
k_{ap}^1	2	k_{ap}^2	2	k_{ap}^3	100
k_{ace}	1				
k_a^0	1	k_a^1	1	k_a^2	1
k_a^3	0.5	k_a^4	1	k_a^5	1
k_a^6	1	k_a^8	1		
k_d^1	0	k_d^2	0		
k_d^4	0	k_d^5	0		
k_d^7	1	k_d^8	0.0001		
k_{ex}^0	5	k_{ex}^1	1	k_{ex}^2	3
k_{ex}^3	1	k_{ex}^4	1	k_{ex}^5	1
k_{im}^0	5	k_{im}^1	1	k_{im}^2	3
k_{im}^3	1	k_{im}^4	1	k_{im}^5	1

Then D_R, or DSB and ROS, will be treated as external control variables for the system of ATM activation. MRN enhances the recruitment of Tip60/ATM to DNA damage site for Tip60 activation and subsequent ATM activation, and stimulates the the substrate recruitment.

References

[1] Bakkenist, C.J., Kastan, M.B.: Dna damage activates atm through intermolecular autophosphorylation and dimer dissociation. Nature **421**(6922), 499–506 (2003)

[2] Sun, Y., Xu, Y., Roy, K., Price, B.D.: Dna damage-induced acetylation of lysine 3016 of atm activates atm kinase activity. Molecular and Cellular Biology **27**(24), 8502–8509 (2007)

[3] Volonte, D., Kahkonen, B., Shapiro, S., Di, Y., Galbiati, F.: Caveolin-1 expression is required for the development of pulmonary emphysema through activation of the atm-p53-p21 pathway. Journal of Biological Chemistry **284**(9), 5462–5466 (2009)
